# Association and biological pathways between lung function and incident depression: a prospective cohort study of 280,032 participants

**DOI:** 10.1186/s12916-024-03382-3

**Published:** 2024-04-15

**Authors:** Wei Hu, Bao-Peng Liu, Cun-Xian Jia

**Affiliations:** Department of Epidemiology, School of Public Health, Cheeloo College of Medicine, Shandong University, Jinan, China

**Keywords:** Lung function, Incident depression, Biomarkers, Mediating mechanisms

## Abstract

**Background:**

Lung health is increasingly recognized as an essential factor in mental health. However, prospective evidence on lung function with incident depression remains to be determined. The study aimed to examine the prospective association between impaired lung function and incident depression and the underlying biological mechanisms.

**Methods:**

This prospective cohort study comprised 280,032 non-depressed individuals with valid lung function measurements from the UK Biobank. Lung function was assessed through the forced vital capacity (FVC) or forced expiratory volume in 1 s (FEV_1_). Cox proportional hazard models were applied to estimate the associations between lung function and incident depression. Mediation analyses were fitted to investigate the potential mediating role of biomarkers and metabolites in the association.

**Results:**

A total of 9514 participants (3.4%) developed depression during a median follow-up of 13.91 years. Individuals in the highest quartile had a lower risk of depression (FVC % predicted: HR = 0.880, 95% CI = 0.830–0.933; FEV_1_% predicted: HR = 0.854, 95% CI = 0.805–0.905) compared with those in the lowest quartile of the lung function indices. Additionally, the restricted cubic splines suggested lung function indices had reversed J-shaped associations with incident depression (nonlinear *P* < 0.05 for FVC % predicted and FEV_1_% predicted). Impaired lung function yielded similar risk estimates (HR = 1.124, 95% CI = 1.074–1.176). Biomarkers involving systemic inflammation, erythrocytes, and liver and renal function may be potential mediators in the lung function-depression association.

**Conclusions:**

This study revealed that the higher risk of developing depression was associated with impaired lung function. Also, the association might be partially mediated by biomarkers including systemic inflammation, erythrocytes, and liver and renal function, though these mediation findings should be interpreted with caution due to potential temporal ambiguity.

**Supplementary Information:**

The online version contains supplementary material available at 10.1186/s12916-024-03382-3.

## Background

Depression, a mental health condition that impacts the well-being of over 300 million individuals globally [1], has emerged as the leading contributor to disability-adjusted life years among mental disorders [2]. Accumulating evidence suggests that depression is associated with a heightened risk of multiple detrimental health outcomes, including all-cause and cancer mortality, as well as cardiovascular disease morbidity and mortality [3, 4]. However, traditional therapies for depression (e.g., medication) are costly and of limited efficacy [5]. Hence, identifying cost-effective strategies to prevent depression is paramount.

Physical health has garnered growing attention as a modifiable protective factor for depression. Whilst research has reported that low grip strength is a significant risk factor for new-onset depression [1], the role of lung function, which is recognized as an inexpensive, non-invasive, and modifiable indicator for assessing physical fitness, in preventing depression remains poorly understood. A review involving 1,161,632 subjects has shed light on the advantages of maintaining moderate or high levels of pulmonary fitness in the prevention of depression [6]. However, it remains unclear whether poor lung function, as measured by forced vital capacity (FVC) and forced expiratory volume in 1 s (FEV_1_), is a direct risk factor for depression. Previous evidence on this topic has primarily focused on either the impact of lung diseases on depression or examined associations in the opposite direction [7, 8]. Whilst prior studies have indicated a lung function-depression association, limitations were exhibited in terms of single sex, small samples, cross-sectional designs, and susceptible populations [9–12].

Existing epidemiological studies have established links between abnormal lung function and various biomarkers, including liver [13], kidney [14], erythrocytes [15], inflammation [16], and circulating metabolites [17]. These biomarkers are also closely associated with the development of depression [18]. For example, impaired lung function may cause cerebral hypoxia and metabolic disturbances [16], which in turn trigger a pro-inflammatory state with the release of large amounts of pro-inflammatory factors that elevate the level of the systemic inflammatory response, resulting in the occurrence of depressive symptoms [8]. Nonetheless, to date, no population-based cohort studies have investigated whether and to what extent these biomarkers may mediate the association between abnormal lung function and depression risks.

To address research gaps, samples from the UK Biobank (UKB) were analyzed to prospectively investigate the association between lung function and incident depression and to delve deeper into the underlying biological mechanisms of this association.

## Methods

### Study population

The UKB, one of the largest prospective cohorts targeting the determinants of a range of complex diseases in middle-aged and older Europeans, recruited over 500,000 participants (aged 37–73) who completed touchscreen questionnaires, physical examination, and biological information at 22 evaluation centers in England, Scotland, and Wales between 2006 and 2010. A comprehensive description of this cohort can be found elsewhere [19]. In short, the cohort aims to provide a detailed investigation of the socio-demographic, lifestyle, environmental, and genetic determinants of a range of complex diseases. Moreover, the cohort ensures continuous tracking of participant morbidity and mortality by establishing links with electronic records from hospitalization and death registries [19].

**Fig. 1:**
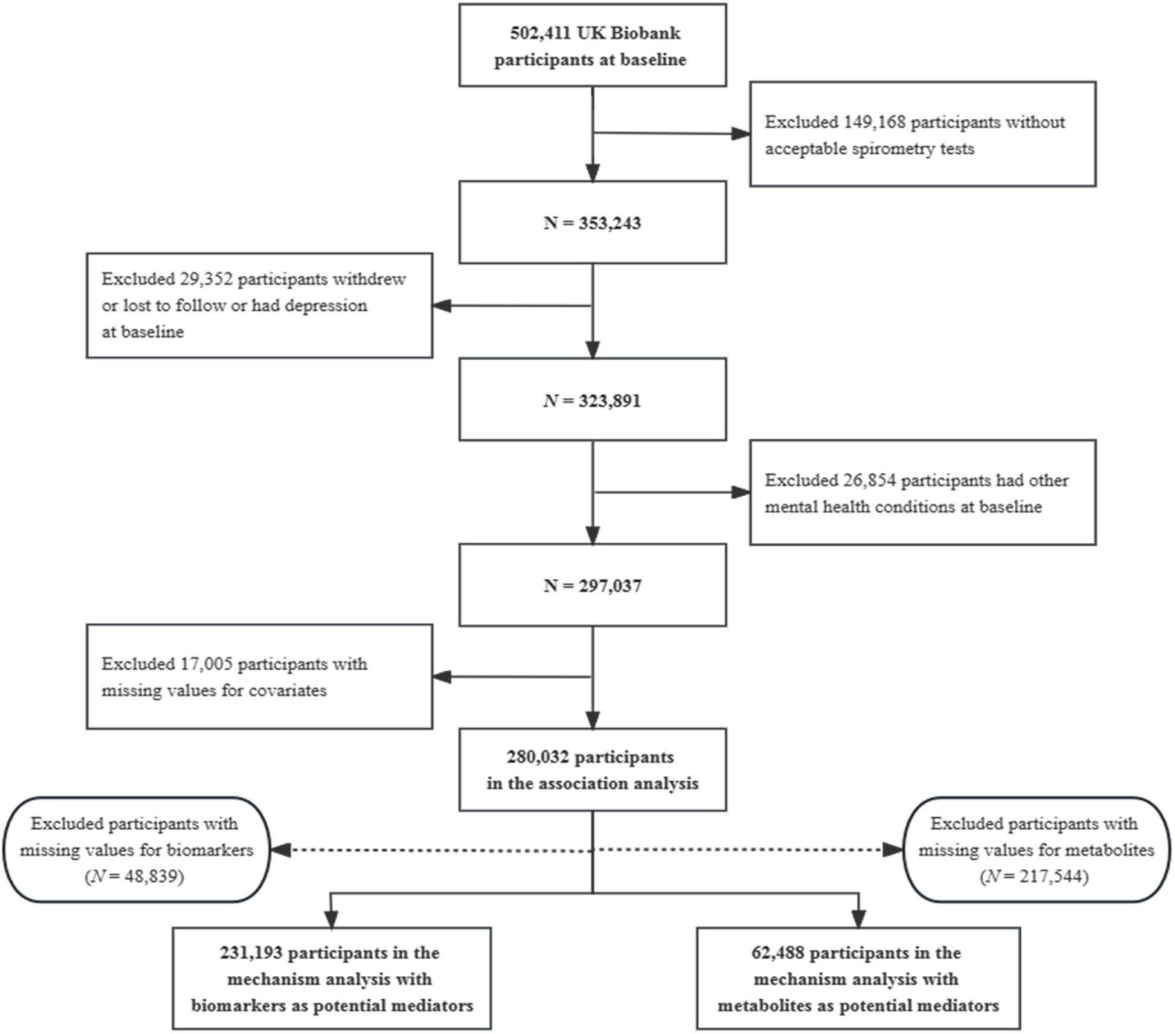
The flow chart of the selection of the study population

Among participants with complete and acceptable lung function data (*N* = 353,243), those with any of the following conditions were also excluded: (1) withdrawal or lost follow-up (*N* = 725); (2) suffering from depression (*N* = 28,537) or other mental symptoms-related disorders (including dementia, anxiety, schizophrenia, bipolar disorder, Parkinson’s disease, Alzheimer’s disease, or substance abuse; *N* = 26,854) at baseline; (3) missing values for covariates (*N* = 17,005). A total of 280,032 participants were included in formal analyses. Also, our study focused on two subpopulations with complete biomarker (*N* = 231,193) or metabolite (*N* = 62,488) information for mediating mechanism exploration. The detailed participant selection process is displayed in Fig. 1.

The authors assert that all procedures contributing to this work comply with the ethical standards of the relevant national and institutional committees on human experimentation and with the Helsinki Declaration of 1975, as revised in 2008.

### Lung function

Trained healthcare professionals utilized a standard spirometer to measure the respiratory function of participants. Participants were instructed to deliver two to three forceful blows within 6 min, with each blow lasting at least 6 s. If the difference between the first two blows was less than 5%, a third blow was not required. We utilized the Global Lung Function Initiative (GLI) 2012 equations based on age, height, sex, and ethnicity to convert the highest values of FVC and FEV_1_ with valid blows into % predicted values for the primary analyses [20]. Participants with FEV_1_ ≥ 80% predicted value and FEV_1_/FVC ≥ 0.70 were considered to possess normal lung function, while the rest had impaired lung function [21].

### Assessment of depression

Depression cases were defined by the International Classification of Diseases, Tenth Revision codes F32 and F33 among the “First occurrence fields”, and were obtained through self-reported medical conditions, primary care, hospital admission, and death registrations [22]. The same methodology was performed to assess the status of depression or other mental disorders at baseline [22]. The date of death for participants from England and Wales was obtained from National Health Service Digital, whereas those for participants from Scotland were sourced from the National Health Service Central Register. The follow-up time was computed as the time interval from the recruitment date to the diagnosis date of depression cases, date of death, or the censoring data, whichever occurred first.

### Assessment of potential mediators

Based on the evidence of potential pathways [13–18], 25 blood biomarkers including inflammation, erythrocytes, liver and renal function, and 168 plasma metabolites were selected as potential mediators. In the UKB, blood tests were performed on participants with informed consent at baseline recruitment. Blood samples of about 4 ml were collected, separated by composition, stored in a refrigerator at−80 °C, and analyzed within 24 h using a Beckman Coulter LH750 instrument (https://biobank.ndph.ox.ac.uk/ukb/ukb/docs/haematology.pdf). The blood biomarkers with rigorous quality checks have undergone external validation (https://biobank.ndph.ox.ac.uk/showcase/showcase/docs/serum_biochemistry.pdf). Plasma samples from about one-fifth of participants in the baseline recruitment were tested for metabolites using Nightingale Health’s NMR-based high-throughput metabolic biomarker analysis platform [23]. According to the online documentation provided by the UKB (https://biobank.ndph.ox.ac.uk/ukb/ukb/docs/nmrm_companion_doc.pdf), a total of 168 metabolites were quantified in molar concentration units, and the metabolite data could be utilized directly for epidemiological analyses without requiring any preprocessing.

Inflammation-related biomarkers included leukocyte count, neutrophil count and percentage, monocyte count and percentage, lymphocyte count and percentage, platelet count, and C reactive protein (CRP). Erythrocyte-related biomarkers suggestive of hypoxia or anemia included erythrocyte count, high light scatter reticulocyte count, reticulocyte count, red blood cell distribution width (RBC), hematocrit percentage, and hemoglobin concentration (HbA1c). Liver function-related biomarkers included alanine aminotransferase (ALT), alkaline phosphatase (ALP), aspartate aminotransferase (AST), gamma-glutamyltransferase (GGT), total bilirubin (TBIL), total protein (TP), and albumin (ALB). Renal function-related biomarkers included cystatin C, urate, and urea. The mean concentration for blood biomarkers and plasma metabolites can be seen in Additional file 1: Tables S1-S2.

### Covariates

A range of factors that may be related to lung function or depression were regarded as covariates [1, 16], including sociodemographic characteristics [(age, sex, height, education level, employment, assessment center, and Townsend deprivation index (TDI)], lifestyles, and medical histories. The TDI covered a wide range of information on social class, employment, and housing and reflected the participant’s area-based socioeconomic status (SES), with higher scores indicating greater deprivation [16]. Smoking status (never, previous or current), drinking status (never, previous or current), physical activity (active vs inactive), body mass index (BMI, continuous), and sleep duration [short (< 7 h), normal (7–8 h) or long (> 8 h)] were adjusted as lifestyle factors. Physical activity was assessed using the International Physical Activity Questionnaire short questionnaire (IPAQ-SQ). Participants were queried regarding the number of days they engaged in more than 10 min of walking, moderate and vigorous physical activity in a week, and the amount of time they engaged in each activity during the day, with 150 min or more of moderate-intensity activity per week defined as physically active [24]. Medical histories (yes vs no) were ascertained through self-reported information and medical records, encompassing hypertension, diabetes, heart failure (HF), stroke, coronary heart disease (CHD), ischemic heart disease (IHD), and lung-related diseases (asthma, chronic obstructive pulmonary disease, emphysema/chronic bronchitis, and other respiratory problems). A summary of missing covariates is provided in Additional file 1: Table S3.

### Statistical analysis

The Wilcoxon tests for continuous variables [mean (SD)] or chi-square tests for categorical variables [frequency (%)] were applied to compare baseline characteristics categorized by incident depression or lung function status. Cox proportional hazard models were utilized to estimate the risk of new-onset depression ascribed to low lung function, with results presented as hazard ratios (HRs) and 95% confidence intervals (CIs). The Cox regression model based on the proportional hazards assumption was suitable for analyzing time-to-event data even if the incidence of the outcome event (depression) was relatively low (less than 5%) in this study, as long as the assumption was met. The proportional hazards assumption was assessed by the Schoenfeld residuals test to check for potential time-variant biases, and we found no correlation between residuals and time (*P* > 0.05) in this study. Despite the low incidence rate, the Cox models make efficient use of all available data by considering both the time until the event and the occurrence of the event, maximizing the information obtained from the cohort. Furthermore, the Cox models allow adjustment for various confounders, which are essential for accurate estimation of risk factor associations.

To explore the association between different levels of lung function and incident depression, we analyzed FVC and FEV_1_ (“rspiro” package in R software) as categorical variables (quartiles), with the lowest quartile (Q1) as the reference group. Dichotomous lung function was employed to examine the longitudinal associations of depression cases with impaired lung function. The restricted cubic spline (“rcssci” package in R software) with four knots was fitted to explore the dose-response relationship between lung function and incident depression. We fitted three Cox models with incremental adjustments for confounders: Model 1 adjusted for sociodemographic covariates; Model 2 additionally accounted for lifestyle factors; Model 3 (priority model) further adjusted for medical histories.

To assess the robustness of results, we conducted several additional analyses. First, we incorporated the cumulative cigarette consumption along with passive smoking duration in Model 3. Cumulative cigarette consumption was quantified as at least 20 cigarettes per day during the year for former or current smokers [16]. Passive smoking duration was calculated as the number of hours per week in the past year of exposure to tobacco smoke from other people in or outside the home [16]. Second, considering the susceptibility of lung function to environmental factors, we further controlled for PM_2.5_ (particulate matter with a diameter of 2.5 µm or less) and NO_2_ (nitrogen dioxide) in Model 3. Third, we excluded individuals with pre-existing lung-related diseases to ascertain the independent impact of lung function. Fourth, to mitigate the potential impact of reverse causality, we eliminated the depression cases occurring within the first 2 years of follow-up. Fifth, covariates with missing values were imputed using the multiple imputations by chained equations method (“mice” package in R software) [25]. Sixth, we further excluded prevalent depression measured by the Patient Health Questionnaire-2 (PHQ-2) at baseline to re-analyze the associations [26]. Stratification analyses were conducted by sex, age, education, employment, TDI, smoking status, drinking status, BMI, and sleep duration to assess potential effect modification. By adding product terms to the Cox models, we used the likelihood ratio tests to examine the interactions of lung function and stratification factors on depression risk.

Selected biomarkers and metabolites could be considered as potential mediators by the following analyses. First, multiple linear regression models were applied to evaluate the association of lung function with biomarkers or metabolites. Second, Cox regression models adjusting for covariates in Model 3 were implemented to examine the relationship between lung function, biomarkers, and incident depression. Biomarkers or metabolites that simultaneously exhibited significance in the aforementioned steps would be considered as potential mediators for subsequent mediation analyses [16, 17, 25]. The proportion mediated (PM) was estimated via the “mediation” package, and the non-parametric bootstrap method (1000 draws) was used to calculate 95% CIs of the PM. Raw data for biomarkers and metabolites were standardized (*z*-score) before entering formal analysis.

All analyses were conducted by SAS 9.4 (SAS Institute, Cary, NC, USA) and R software (4.0.5). In analyses concerning biomarkers or metabolites, a false discovery rate (FDR) adjusted *P* < 0.05 was regarded as statistically significant [27]. In the prospective association analysis, a two-tailed *P* < 0.05 was regarded as statistically significant.

## Results

Of the sample of 280,032 in the association analysis, 147,923 (52.8%) were female, with a mean age (SD) of 56.5 (8.0) years. During more than 3,759,362 person-years of follow-up (median follow-up: 13.91 years), 3.4% of participants without depression or other psychiatric disorders at baseline developed depression. In comparison to the healthy controls, depressed patients were more likely to be female, be less educated, be unemployed, be in a poorer SES situation, smoke but do not regularly drink alcohol, be physically inactive, have a higher BMI, have an abnormal sleep duration, and have a lower FEV_1_ and FVC (*P* < 0.001) (Table 1). When grouped by lung function status, we observed similar baseline characteristics (Additional file 1: Table S4).

**Table 1:** Baseline characteristics by depression status

**Characteristics**	**Overall**	**Incident depression**		***P* value**
	**(*N* = 280,032)**	**No**	**Yes**	
		**(*N* = 270,518)**	**(*N* = 9514)**	
Age, mean (SD)	56.5 (8.0)	56.5 (8.0)	56.0 (8.4)	< 0.001
Sex,* n* (%)				< 0.001
Female	147,923 (52.8)	142,069 (52.5)	5854 (61.5)	
Male	132,109 (47.2)	128,449 (47.5)	3660 (38.5)	
Education, *n* (%)				< 0.001
College or university	96,896 (34.6)	94,351 (34.9)	2545 (26.8)	
Others	183,136 (65.4)	176,167 (65.1)	6969 (73.2)	
Employment, *n* (%)				< 0.001
Employed	171,392 (61.2)	166,012 (61.4)	5380 (56.5)	
Non-employed	108,640 (38.8)	104,506 (38.6)	4134 (43.5)	
Townsend deprivation index, mean (SD)	−1.6 (2.9)	−1.7 (2.9)	−1.0 (3.2)	< 0.001
Assessment centers, *n* (%)				< 0.001
England	218,065 (77.9)	210,703 (77.9)	7362 (77.4)	
Scotland	29,409 (10.5)	28,584 (10.6)	825 (8.7)	
Wales	32,558 (11.6)	31,231 (11.5)	1327 (13.9)	
Smoking status, *n* (%)				< 0.001
Never	157,149 (56.1)	152,547 (56.4)	4602 (48.4)	
Previous	100,561 (35.9)	96,916 (35.8)	3645 (38.3)	
Current	22,322 (8.0)	21,055 (7.8)	1267 (13.3)	
Drinking status, *n* (%)				< 0.001
Never	7906 (2.8)	7561 (2.8)	345 (3.6)	
Previous	7446 (2.7)	6961 (2.6)	485 (5.1)	
Current	264,680 (94.5)	255,996 (94.6)	8684 (91.3)	
Physical activity, *n* (%)				< 0.001
Physically active	146,902 (52.5)	142,396 (52.6)	4506 (47.4)	
Physically inactive	133,130 (47.5)	128,122 (47.4)	5008 (52.6)	
BMI, mean (SD)	27.2 (4.6)	27.2 (4.5)	28.3 (5.4)	< 0.001
Sleep duration (%)				
Normal (7–8 h)	196,959 (70.3)	191,194 (70.7)	5765 (60.6)	< 0.001
Short (< 7 h)	65,006 (23.2)	62,111 (22.9)	2895 (30.4)	
Long (> 8 h)	18,067 (6.5)	17,213 (6.4)	854 (9.0)	
Hypertension, *n* (%)	69,215 (24.7)	66,282 (24.5)	2933 (30.8)	< 0.001
Diabetes, *n* (%)	11,483 (4.1)	10,852 (4.0)	631 (6.6)	< 0.001
Stroke, *n* (%)	4814 (1.7)	4523 (1.7)	291 (3.1)	< 0.001
Coronary heart disease, *n* (%)	12,100 (4.3)	11,439 (4.2)	661 (7.0)	< 0.001
Heart failure, *n* (%)	1063 (0.4)	1016 (0.4)	47 (0.5)	0.065
Ischemic heart disease, *n* (%)	7133 (2.6)	6744 (2.5)	389 (4.1)	< 0.001
Lung-related diseases, *n* (%)	35,501 (12.7)	33,746 (12.5)	1755 (18.5)	< 0.001
FEV_1_% predicted, mean (SD)	93.4 (16.4)	93.5 (16.3)	90.6 (17.2)	< 0.001
FVC % predicted, mean (SD)	97.1 (15.2)	97.2 (15.2)	95.0 (15.9)	< 0.001

*BMI* Body mass index, *SD* Standard deviation, *FEV*_*1*_ Forced expiratory volume in 1 s, *FVC* Forced vital capacity

**Fig. 2:**
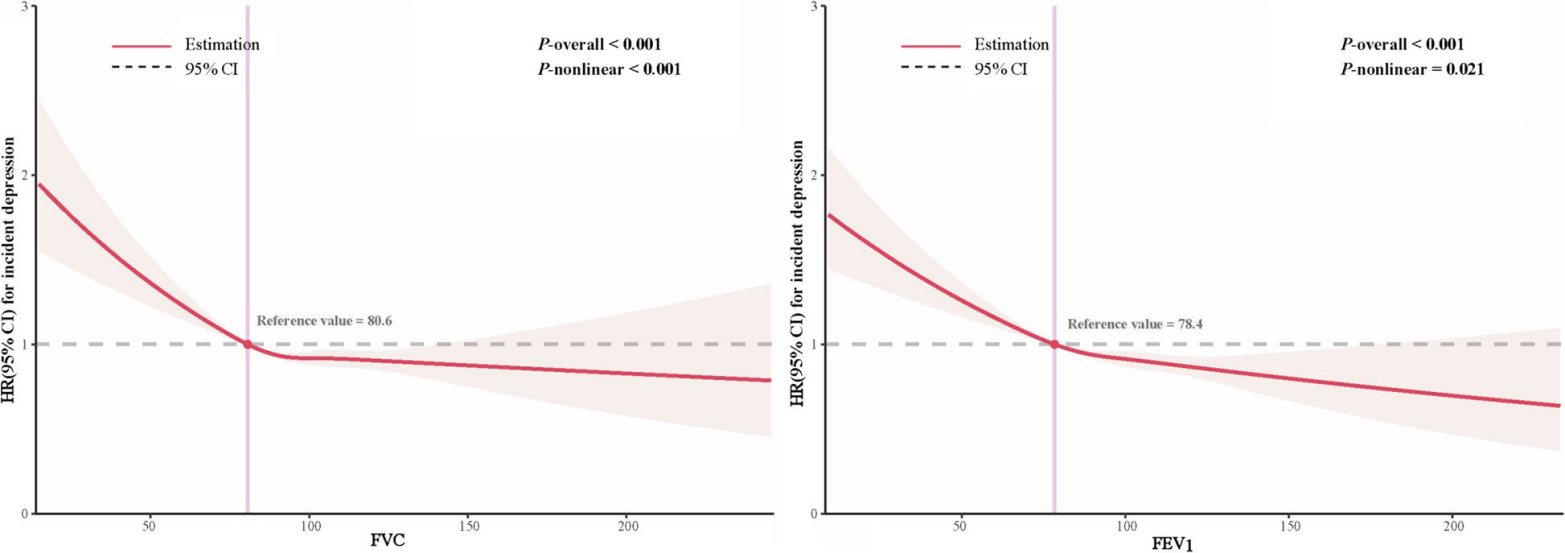
Restricted cubic spline analyses for the association of FVC (% predicted) and FEV_1_ (% predicted) with incident depression

We observed a non-linear dose-response relationship between lung function and risk of depression (nonlinear *P* < 0.05 for FVC and FEV_1_) (Fig. 2). The prospective lung function-depression association is displayed in Table 2. We observed a significant protective role of lung function indices for depression risks in the fully adjusted models (model 3). For FVC (% predicted), the HRs of depression were 0.874 (95% CI = 0.827–0.924; *P* < 0.001), 0.888 (95% CI = 0.839–0.940; *P* < 0.001), and 0.880 (95% CI = 0.830–0.933; *P* < 0.001) in quartiles 2 to 4 compared with the lowest quartile. For FEV_1_ (% predicted), the HRs of depression were 0.903 (95% CI = 0.855–0.954; *P* < 0.001), 0.860 (95% CI = 0.813–0.911; *P* < 0.001), and 0.854 (95% CI = 0.805–0.905; *P* < 0.001) in quartiles 2 to 4 compared with the lowest quartile. Additionally, individuals with impaired lung function had a 12.4% (HR = 1.124, 95% CI = 1.074–1.176) increased risk of developing depression compared to those with normal lung function. Several sensitivity analyses did not affect the robustness of the findings (Additional file 1: Tables S5-S8). Stratification analysis discovered that detrimental effects of impaired lung function were stronger among individuals who were older, less educated, non-employed, living in areas with high TDI, and current or former smokers (*P*_for interaction_ < 0.05) (Additional file 1: Fig. S1).

**Table 2:** Association between lung function and risk of incident depression

**Variables**	**No. of depression/person-year**	**Risk of incident depression**					
		**Model 1**		**Model 2**		**Model 3**	
		**HR (95% CI)**	***P* value**	**HR (95% CI)**	***P* value**	**HR (95% CI)**	***P* value**
**FVC (% predicted)**							
Quartile 1 (lowest)	2948/923,244	1 (Reference)		1 (Reference)		1 (Reference)	
Quartile 2	2285/940,455	0.783 (0.741–0.827)	< 0.001	0.843 (0.797–0.890)	< 0.001	0.874 (0.827–0.924)	< 0.001
Quartile 3	2193/944,912	0.755 (0.714–0.798)	< 0.001	0.845 (0.799–0.894)	< 0.001	0.888 (0.839–0.940)	< 0.001
Quartile 4 (highest)	2088/950,751	0.713 (0.674–0.754)	< 0.001	0.828 (0.781–0.877)	< 0.001	0.880 (0.830–0.933)	< 0.001
**FEV**_**1**_** (% predicted)**							
Quartile 1 (lowest)	2982/921,978	1 (Reference)		1 (Reference)		1 (Reference)	
Quartile 2	2378/940,557	0.805 (0.762–0.850)	< 0.001	0.861 (0.815–0.909)	< 0.001	0.903 (0.855–0.954)	0.001
Quartile 3	2143/946,846	0.732 (0.692–0.774)	< 0.001	0.807 (0.763–0.854)	< 0.001	0.860 (0.813–0.911)	< 0.001
Quartile 4 (highest)	2011/949,980	0.695 (0.656–0.735)	< 0.001	0.789 (0.745–0.836)	< 0.001	0.854 (0.805–0.905)	< 0.001
**Lung function**							
Normal	6635/2,825,766	1 (Reference)		1 (Reference)		1 (Reference)	
Impaired	2879/933,595	1.289 (1.233–1.347)	< 0.001	1.195 (1.142–1.249)	< 0.001	1.124 (1.074–1.176)	< 0.001

Model 1 adjusted for age at baseline, sex, education, employment, assessment center, Townsend Deprivation Index, and height

Model 2 adjusted for model 1 plus smoking status, drinking status, physical activity, BMI, and sleep duration

Model 3 adjusted for model 2 plus hypertension, diabetes, heart failure, stroke, coronary heart disease, ischemic heart disease, and pre-existing lung diseases (yes or no)

*HR* Hazards ratio, *CI* Confidence interval, *FEV*_*1*_ Forced expiratory volume in 1 s, *FVC* Forced vital capacity

Although impaired lung function was substantially associated with all selected biomarkers except monocyte percentage, hematocrit percentage, urea, and ALT, only some of them were significant predictors of depression risk, such as leukocyte count, neutrophil count and percentage, lymphocyte count and percentage, CRP, platelet count, erythrocyte count, RBC distribution width, hemoglobin concentration, cystatin C, urate, ALP, AST, GGT, TBIL, TP, and ALB (*FDR* < 0.05) (Additional file 1: Table S9). Hence, these biomarkers may serve as potential mediators from impaired lung function to incident depression. Additionally, lung function was significantly associated with most metabolites, but none of these metabolites were statistically associated with depression risk (Additional file 1: Table S10). Therefore, no metabolites are available as potential mediators of the association between impaired lung function and incident depression.

**Fig. 3:**
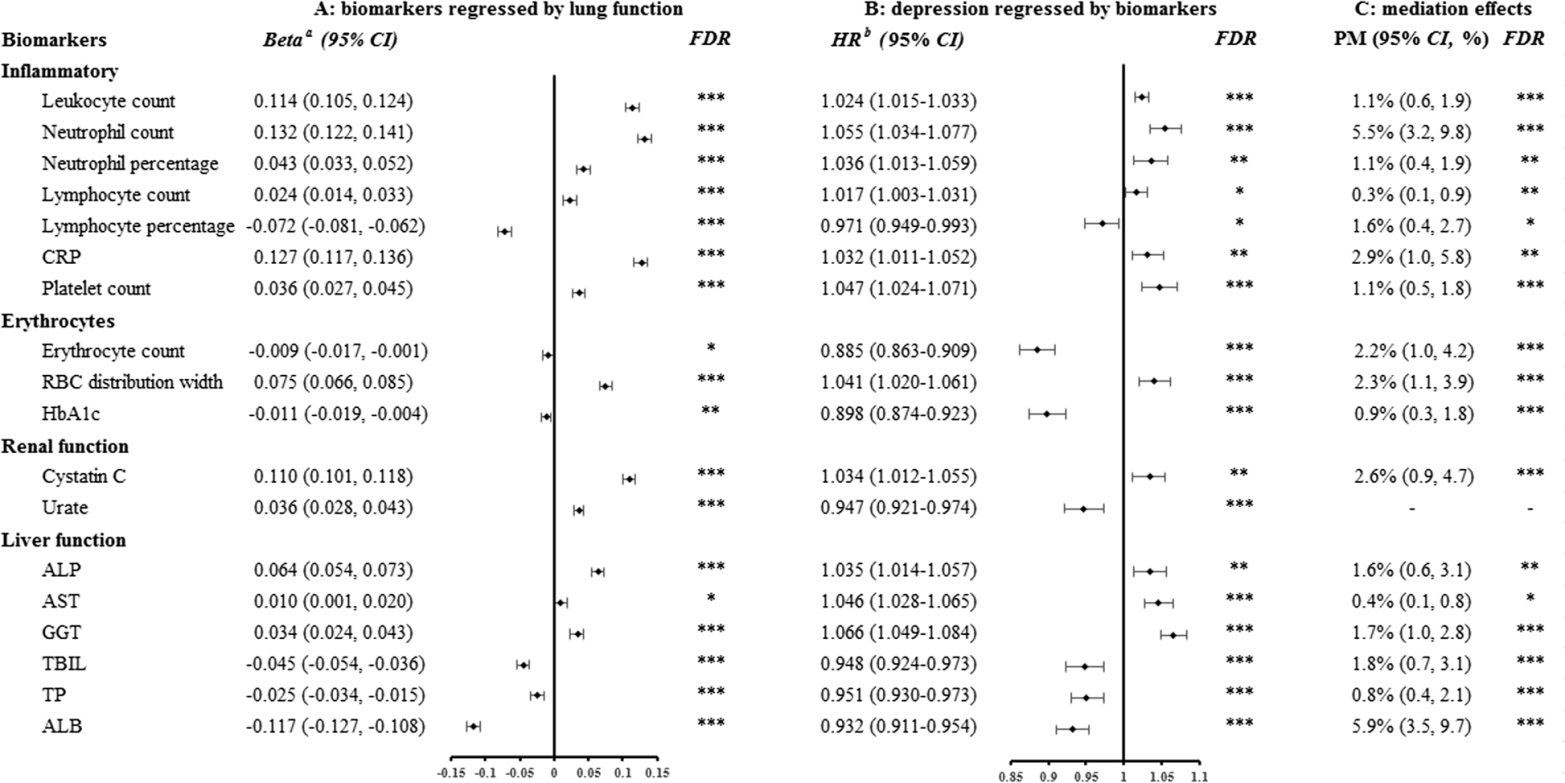
Association of lung function with incident depression mediated by biomarkers

Figure 3 exhibits the PM of these potential mediators that were simultaneously associated with both lung function and incident depression. We observed significant mediating effects of inflammation, suggesting that impaired lung function might increase the risk of depression through the induction of inflammatory dysregulation (*FDR* < 0.05). Neutrophil count and CRP might explain 5.5% (95% CI = 3.2–9.8%) and 2.9% (1.0–5.8%) of the association, respectively. Markers related to erythrocytes and renal function might partially account for the increased risk of depression caused by impaired lung function. The overall effect of low lung function leading to an increased risk of depression might be partially explained by biomarkers related to liver function, with PM (95% CI) ranging from 0.4 (0.1–0.8%) to 5.9% (3.5–9.7%).

## Discussion

Based on a large prospective cohort with a median follow-up of exceeding 13 years, our findings indicated that participants with impaired lung function had a higher risk of depression, independent of confounders including sociodemographic, lifestyle, environment, and comorbidities. The nonlinear dose-response curves revealed that the risk of new-onset depression was steeper at low lung function. Four biomarker pathways, including inflammation, erythrocytes, liver, and renal function, but not metabolite pathways, might partially mediate the association between impaired lung function and incident depression.

Supporting our findings, prior studies have documented a negative association between lung function and depression, but mostly using small samples, cross-sectional designs, or implemented in susceptible populations. A cross-sectional study from the National Health and Nutrition Examination Survey (NHANES) revealed a significant negative FVC-depression association [11]. Another cross-sectional survey based on the NHANES also reported an association between impaired lung function and self-reported poor mental health (including depression) [12]. Moreover, a study including 121 silicosis patients suggested that low FEV_1_ and FVC were cross-sectional associated with depressive symptoms [10]. Notably, Mutz et al., taking advantage of a cross-sectional design, found that lung function in male patients with depression was poorer compared to that of healthy controls, aligning with our findings [28]. However, the opposite was observed in females [28], which might be attributed to the protective effect of estrogen on the lungs [29], shielding them from the detrimental impact of depression. To our awareness, only two studies employed a longitudinal design to investigate the relationship between lung function and incident depression, but they focused on lung disease or were conducted in single-sex populations exclusively. For instance, a longitudinal study enrolling 10,508 Chinese adults with an average follow-up period of 3 years reported that participants with chronic lung disease (CLDs) at baseline had a higher risk of depressive symptoms [7]. Another prospective study of 1205 middle-aged men revealed that low respiratory function was associated with an increased risk of subsequent depressive symptoms [9]. Remarkably, the aforementioned studies predominantly relied on self-report measures for the identification of depression rather than clinical diagnosis. Additionally, a previous UK Biobank study using Mendelian randomization (MR) provided evidence of a causal effect of depression on outcomes of impaired lung function, such as asthma [30]. Combined with our findings, we speculated that there may be a reciprocal causal relationship between lung function and depression. However, to our knowledge, no extant studies have provided evidence of a causal effect of lung function on depression. Based on a large population with long-term follow-up, the study elucidated for the first time the negative associations of lung function with incident depression through adequate adjustment for confounders. Although still observational, our findings may provide insights into causal effects. Future studies are warranted to validate the causality of our findings.

Prior investigations examining the relationship between lung function and adverse outcomes have demonstrated a significant nonlinear association [31, 32]. Our study substantiated this and furnished original evidence for the nonlinear association between lung function indicators and depression. Stratified analyses suggested that the lung function-depression association was modified by several sociodemographic and lifestyle factors. First, cigarette consumption exacerbated the negative impact of impaired lung function on depression, which was similar to the trend reported in previous research [7]. Specifically, although no significant interactions were found, the trend found by Ren et al. that the adverse effect of CLDs at baseline on incident depression was stronger in participants who were current smokers was similar to our findings [7]. One possible explanation is that the noxious gases and particulate matter generated during tobacco combustion may cause airflow obstruction, which is a primary cause of depression owing to impaired lung function [33]. Furthermore, the deleterious effects of poor lung function on subsequent depressive symptoms were pronounced in populations with low SES, which was congruent with a previous survey that concluded that individuals with low SES experience a reduction in the beneficial impact of improved lung function on overall health [34].

Our first exploratory analyses examining potential mediating mechanisms contributed to a deeper understanding of the heightened risk of depression associated with impaired lung function. First, we observed that several inflammatory factors, such as leukocyte count, neutrophil count and percentage, lymphocyte count and percentage, CRP, and platelet, might partially explain the detrimental effects of impaired lung function, which was in accordance with previous observational studies [35–37]. Impaired lung function can lead to excessive release of pro-inflammatory cytokines [35, 36], resulting in elevated plasma levels of glucocorticoids and subsequently contributing to the onset of depression [37]. Second, erythrocyte-associated markers hinting at hypoxia and anemia (including RBC and HbA1c) may mediate, to some extent, the lung function-depression association, supporting the previously hypothesized underlying pathological mechanisms of hypoxemia and hypercapnia [11]. Specifically, low lung function that disrupts the exchange of oxygen and carbon dioxide could engender both conditions [38], causing metabolic dysregulation in brain cells and ultimately triggering depressive symptoms [39]. Moreover, the increased risk of depression ascribed to impaired lung function might be partially explained by the deterioration in the liver and renal functions, in line with prior studies [13, 14, 40, 41]. For instance, a cohort study involving more than 370,000 participants revealed a significant association between liver function markers, such as ALT, TBIL, ALB, TP, GGT, and ALP, and an elevated risk of lung disease [13]. Also, a MR study supported a causal relationship between lung diseases and renal function [14]. Remarkably, the relationship between liver and renal dysfunction and depression has been well established [40, 41].

The main strength of this study was the comprehensive examination of the prospective lung function-depression association and the preliminary exploration of the underlying biological mechanisms of the association. Other strengths comprised large sample sizes, long follow-up, sufficient adjustment for confounders, reliable methods for assessing lung function, and identification of depression from multiple sources including clinical diagnosis.

Several limitations should be considered. First, the participants in the cohort were overwhelmingly white, limiting the extrapolation of our findings. Second, most of the confounders are self-reported, which can introduce recall bias. Third, we did not find any statistical association between metabolites and incident depression cases, which may be due to the low rate of incident depression. Fourth, the methods we used to identify cases of depression may have insufficient power in identifying mild cases, which could lead to misclassification bias and thus obscure the associations found by the study. Nevertheless, sensitivity analyses after excluding possible cases of mild depression additionally identified by PHQ-2 at baseline did not substantially change the robustness of the associations. Fifth, although the UK Biobank cohort has a “healthy volunteer” bias that may affect the representativeness of our findings [42], the associations of risk factors in the cohort have been demonstrated to be similar to those found in the representative cohort [42]. Sixth, although we excluded the participants with a history of depression at baseline, the age of onset of depression is usually earlier than the lower age limit (37 years) of the UK Biobank sample, so caution is necessary in applying our findings to a younger population. Seventh, despite our efforts to control for a range of confounders, residual confounding may still exist. Eighth, the inherent nature of observational studies precludes us from drawing causal inferences. Further MR or clinical trials are warranted to confirm causality. Additionally, the mediation analysis method used in this study could only assess one mediator at a time to explore the single pathway of mediating mechanisms for associations between lung function and incident depression. Multiple pathways adopting serial mediation or structural equation model are needed in further studies. Finally, longitudinal mediation analyses necessitate a clear temporal relationship between variables, but the exposure and mediators in the present study were measured at the same time point due to data availability constraints. Nonetheless, the previously proposed lung-brain-axis hypothesis, which suggests that impaired lung function can affect brain structure via the blood pathway, may theoretically support the temporal relationship between the exposures and mediators in our study [43]. Caution is warranted in interpreting the mediation results, and future studies with well-defined temporal relationships are required to validate the mediating role of these markers.

## Conclusions

Based on a large-scale prospective cohort, the study revealed that impaired lung function was associated with an increased risk of developing depression. Regular screening of lung function in routine practice has the potential to facilitate the identification of at-risk populations and the development of personalized interventions, yielding profound clinical and public health implications. Additionally, our findings underscored that the biomarkers involving systemic inflammation, erythrocytes, and liver and renal function may partially mediate this association, but these mediation findings should be interpreted with caution due to potential temporal ambiguity. Further studies with repeatedly measured data are warranted to replicate our mediation findings.

### Supplementary Information

**Additional file 1: Table S1.** Mean concentration of biomarkers (*n *= 231,193). **Table S2.** Mean concentration of metabolites (*n* = 62,488). **Table S3.** Summary of missing data of covariates. **Table S4.** Baseline characteristics by lung function (*n* = 280,032). **Table S5.** Sensitivity analyses for association between incident depression and lung function with further adjustment for covariates. **Table S6.** Sensitivity analyses for association between incident depression and lung function with different exclusion criteria. **Table S7.** Sensitivity analyses for association between lung function and risk of incident depression with multiple imputation for missing covariates (*n *= 297,037). **Table S8.** Sensitivity analyses for association between lung function and risk of incident depression with further excluding prevalent depression as measured by the PHQ-2 scale at baseline (*n* = 271,122). **Table S9.** Selection of biomarkers as potential mediators between lung function and incident depression (*n *= 231,193). **Table S10.** Selection of metabolites as potential mediators between lung function and incident depression (*n* = 62,488). **Fig. S1.** Subgroup analysis of the association of lung function on depression by potential risk factors.

## Data Availability

Data supporting the results of this study are available on request from the UK Biobank team (http://www.ukbiobank.ac.uk/).

## Abbreviations

ALB: Albumin
ALP: Alkaline phosphatase
ALT: Alanine aminotransferase
AST: Aspartate aminotransferase
CIs: Confidence intervals
CLDs: Chronic lung disease
CRP: C reactive protein
FEV_1_: Forced expiratory volume in 1 s
FVC: Forced vital capacity
GGT: Gamma-glutamyltransferase
GLI: Global Lung Function Initiative
HbA1c: Hemoglobin concentration
HRs: Hazard ratios
NO_2_: Nitrogen dioxide
PM: Proportion mediated
PM_2.5_: Particulate matter with a diameter of 2.5 µm or less
RBC: Red blood cell
SES: Socioeconomic status
TBIL: Total bilirubin
TDI: Townsend deprivation index
TP: Total protein
UKB: UK Biobank
