# Supplementary material for: Association and biological pathways between lung function and incident depression: a prospective cohort study of 280,032 participants
Source: BMC Med. 2024 Apr 15;22:160. doi: 10.1186/s12916-024-03382-3 (PMC11017623; doi:10.1186/s12916-024-03382-3)
Supplement: Supplementary file 1 — Additional file 1: Table S1. Mean concentration of biomarkers (n = 231,193). Table S2. Mean concentration of metabolites (n = 62,488). Table S3. Summary of missing data of covariates. Table S4. Baseline characteristics by lung function (n = 280,032). Table S5. Sensitivity analyses for association between incident depression and lung function with further adjustment for covariates. Table S6. Sensitivity analyses for association between incident depression and lung function with different exclusion criteria. Table S7. Sensitivity analyses for association between lung function and risk of incident depression with multiple imputation for missing covariates (n = 297,037). Table S8. Sensitivity analyses for association between lung function and risk of incident depression with further excluding prevalent depression as measured by the PHQ-2 scale at baseline (n = 271,122). Table S9. Selection of biomarkers as potential mediators between lung function and incident depression (n = 231,193). Table S10. Selection of metabolites as potential mediators between lung function and incident depression (n = 62,488). Fig. S1. Subgroup analysis of the association of lung function on depression by potential risk factors. [file 12916_2024_3382_MOESM1_ESM.docx]

**Additional file 1**

[**Table S1.** Mean concentration of biomarkers (*n* = 231,193) 2](#_Toc163345822)

[**Table S2.** Mean concentration of metabolites (*n* = 62,488) 3](#_Toc163345823)

[**Table S3**. Summary of missing data of covariates 7](#_Toc163345824)

[**Table S4.** Baseline characteristics by lung function (*n* = 280,032) 8](#_Toc163345825)

[**Table S5.** Sensitivity analyses for association between incident depression and lung function with further adjustment for covariates 9](#_Toc163345826)

[**Table S6.** Sensitivity analyses for association between incident depression and lung function with different exclusion criteria 10](#_Toc163345828)

[**Table S7.** Sensitivity analyses for association between lung function and risk of incident depression with multiple imputation for missing covariates (*n* = 297,037) 11](#_Toc163345830)

[**Table S8.** Sensitivity analyses for association between lung function and risk of incident depression with further excluding prevalent depression as measured by the PHQ-2 scale at baseline (*n* = 271,122) 12](#_Toc163345832)

[**Table S9.** Selection of biomarkers as potential mediators between lung function and incident depression (*n* = 231,193) 13](#_Toc163345833)

[**Table S10**. Selection of metabolites as potential mediators between lung function and incident depression (*n* = 62,488) 14](#_Toc163345834)

[**Fig. S1.** Subgroup analysis of the association of lung function on depression by potential risk factors. 18](#_Toc163345835)

**Table S1.** Mean concentration of biomarkers (*n* = 231,193)

| **Biomarkers** | **Unit** | **Mean** | **SD** |
| --- | --- | --- | --- |
| **Inflammatory-related biomarkers** |  |  |  |
| Leukocyte count | 10^9 cells/L | 6.79 | 2.14 |
| Neutrophil count | 10^9 cells/L | 4.17 | 1.36 |
| Neutrophil percentage | % | 60.89 | 8.30 |
| Monocyte count | 10^9 cells/L | 0.47 | 0.32 |
| Monocyte percentage | % | 7.12 | 2.62 |
| Lymphocyte count | 10^9 cells/L | 1.94 | 1.20 |
| Lymphocyte percentage | % | 28.87 | 7.28 |
| C reactive protein | mg/L | 2.37 | 4.00 |
| Platelet count | 10^9 cells/L | 251.87 | 58.53 |
| **Erythrocyte-related biomarkers** |  |  |  |
| Erythrocyte count | 10^12 cells/L | 4.53 | 0.41 |
| High light scatter reticulocyte count | 10^12 cells/L | 0.02 | 0.01 |
| Reticulocyte count | 10^12 cells/L | 0.06 | 0.04 |
| Red blood cell distribution width | % | 13.44 | 0.92 |
| Haematocrit percentage | % | 41.25 | 3.48 |
| Haemoglobin concentration | g/dL | 14.25 | 1.23 |
| **Renal function-related biomarkers** |  |  |  |
| Cystatin C | mg/L | 0.90 | 0.16 |
| Urate | μmol/L | 79.62 | 89.10 |
| Urea | mmol/L | 5.43 | 1.34 |
| **Liver function-related biomarkers** |  |  |  |
| Alanine aminotransferase | U/L | 23.50 | 13.85 |
| Alkaline phosphatase | U/L | 82.29 | 25.14 |
| Aspartate aminotransferase | U/L | 26.11 | 9.67 |
| Gamma-glutamyl transferase | U/L | 36.34 | 38.81 |
| Total bilirubin | μmol/L | 9.31 | 4.47 |
| Total protein | g/L | 72.38 | 3.99 |
| Albumin | g/L | 45.34 | 2.58 |

**Table S2.** Mean concentration of metabolites (*n* = 62,488)

| **Metabolites** | **Mean** | **SD** |
| --- | --- | --- |
| Total Cholesterol, mmol/L | 4.59 | 0.92 |
| Total Cholesterol Minus HDL-C, mmol/L | 3.28 | 0.82 |
| Remnant Cholesterol, mmol/L | 1.56 | 0.41 |
| VLDL Cholesterol, mmol/L | 0.72 | 0.24 |
| Clinical LDL Cholesterol, mmol/L | 2.53 | 0.72 |
| LDL Cholesterol, mmol/L | 1.72 | 0.43 |
| HDL Cholesterol, mmol/L | 1.31 | 0.32 |
| Total Triglycerides, mmol/L | 1.29 | 0.56 |
| Triglycerides in VLDL, mmol/L | 0.91 | 0.47 |
| Triglycerides in LDL, mmol/L | 0.14 | 0.04 |
| Triglycerides in HDL, mmol/L | 0.14 | 0.05 |
| Total Phospholipids in Lipoprotein Particles, mmol/L | 2.91 | 0.46 |
| Phospholipids in VLDL, mmol/L | 0.47 | 0.18 |
| Phospholipids in LDL, mmol/L | 0.60 | 0.14 |
| Phospholipids in HDL, mmol/L | 1.54 | 0.31 |
| Total Esterified Cholesterol, mmol/L | 3.34 | 0.66 |
| Cholesteryl Esters in VLDL, mmol/L | 0.43 | 0.14 |
| Cholesteryl Esters in LDL, mmol/L | 1.26 | 0.31 |
| Cholesteryl Esters in HDL, mmol/L | 1.02 | 0.25 |
| Total Free Cholesterol, mmol/L | 1.26 | 0.26 |
| Free Cholesterol in VLDL, mmol/L | 0.29 | 0.11 |
| Free Cholesterol in LDL, mmol/L | 0.46 | 0.12 |
| Free Cholesterol in HDL, mmol/L | 0.29 | 0.07 |
| Total Lipids in Lipoprotein Particles, mmol/L | 8.79 | 1.61 |
| Total Lipids in VLDL, mmol/L | 2.10 | 0.85 |
| Total Lipids in LDL, mmol/L | 2.47 | 0.59 |
| Total Lipids in HDL, mmol/L | 2.99 | 0.63 |
| Total Concentration of Lipoprotein Particles, mmol/L | 0.017 | 0.002 |
| Concentration of VLDL Particles, mmol/L | 0.00014 | 0.00004 |
| Concentration of LDL Particles, mmol/L | 0.0012 | 0.0003 |
| Concentration of HDL Particles, mmol/L | 0.015 | 0.002 |
| Average Diameter for VLDL Particles, nm | 38.61 | 1.25 |
| Average Diameter for LDL Particles, nm | 23.92 | 0.09 |
| Average Diameter for HDL Particles, nm | 9.65 | 0.21 |
| Phosphoglycerides, mmol/L | 2.26 | 0.39 |
| Total Cholines, mmol/L | 2.54 | 0.40 |
| Phosphatidylcholines, mmol/L | 2.08 | 0.37 |
| Sphingomyelins, mmol/L | 0.44 | 0.07 |
| Apolipoprotein B, g/l | 0.84 | 0.20 |
| Apolipoprotein A1, g/l | 1.44 | 0.24 |
| Total Fatty Acids, mmol/L | 11.83 | 2.34 |
| Degree of Unsaturation, degree | 1.36 | 0.08 |
| Omega-3 Fatty Acids, mmol/L | 0.53 | 0.22 |
| Omega-6 Fatty Acids, mmol/L | 4.46 | 0.67 |
| Polyunsaturated Fatty Acids, mmol/L | 4.99 | 0.79 |
| Monounsaturated Fatty Acids, mmol/L | 2.80 | 0.80 |
| Saturated Fatty Acids, mmol/L | 4.04 | 0.93 |
| Linoleic Acid, mmol/L | 3.42 | 0.67 |
| Docosahexaenoic Acid, mmol/L | 0.24 | 0.08 |
| Alanine, mmol/L | 0.30 | 0.08 |
| Glutamine, mmol/L | 0.53 | 0.08 |
| Glycine, mmol/L | 0.16 | 0.06 |
| Histidine, mmol/L | 0.06 | 0.01 |
| Total Concentration of Branched-Chain Amino Acids, mmol/L | 0.35 | 0.08 |
| Isoleucine, mmol/L | 0.05 | 0.02 |
| Leucine, mmol/L | 0.10 | 0.03 |
| Valine, mmol/L | 0.20 | 0.04 |
| Phenylalanine, mmol/L | 0.05 | 0.01 |
| Tyrosine, mmol/L | 0.06 | 0.01 |
| Glucose, mmol/L | 3.54 | 1.05 |
| Lactate, mmol/L | 3.81 | 1.06 |
| Pyruvate, mmol/L | 0.08 | 0.03 |
| Citrate, mmol/L | 0.06 | 0.01 |
| 3-Hydroxybutyrate, mmol/L | 0.06 | 0.06 |
| Acetate, mmol/L | 0.02 | 0.01 |
| Acetoacetate, mmol/L | 0.01 | 0.01 |
| Acetone, mmol/L | 0.01 | 0.01 |
| Creatinine, mmol/L | 0.07 | 0.01 |
| Albumin, g/l | 39.12 | 3.25 |
| Glycoprotein Acetyls, mmol/L | 0.78 | 0.11 |
| Concentration of Chylomicrons and Extremely Large VLDL Particles, mmol/L | 1.69e-6 | 1.50e-6 |
| Total Lipids in Chylomicrons and Extremely Large VLDL, mmol/L | 0.23 | 0.20 |
| Phospholipids in Chylomicrons and Extremely Large VLDL, mmol/L | 0.04 | 0.03 |
| Cholesterol in Chylomicrons and Extremely Large VLDL, mmol/L | 0.06 | 0.04 |
| Cholesteryl Esters in Chylomicrons and Extremely Large VLDL, mmol/L | 0.03 | 0.02 |
| Free Cholesterol in Chylomicrons and Extremely Large VLDL, mmol/L | 0.03 | 0.02 |
| Triglycerides in Chylomicrons and Extremely Large VLDL, mmol/L | 0.14 | 0.13 |
| Concentration of Very Large VLDL Particles, mmol/L | 3.46e-6 | 2.24e-6 |
| Total Lipids in Very Large VLDL, mmol/L | 0.20 | 0.13 |
| Phospholipids in Very Large VLDL, mmol/L | 0.04 | 0.03 |
| Cholesterol in Very Large VLDL, mmol/L | 0.05 | 0.03 |
| Cholesteryl Esters in Very Large VLDL, mmol/L | 0.03 | 0.01 |
| Free Cholesterol in Very Large VLDL, mmol/L | 0.02 | 0.01 |
| Triglycerides in Very Large VLDL, mmol/L | 0.11 | 0.08 |
| Concentration of Large VLDL Particles, mmol/L | 1.02e-6 | 5.45e-6 |
| Total Lipids in Large VLDL, mmol/L | 0.33 | 0.17 |
| Phospholipids in Large VLDL, mmol/L | 0.07 | 0.04 |
| Cholesterol in Large VLDL, mmol/L | 0.10 | 0.05 |
| Cholesteryl Esters in Large VLDL, mmol/L | 0.05 | 0.02 |
| Free Cholesterol in Large VLDL, mmol/L | 0.05 | 0.02 |
| Triglycerides in Large VLDL, mmol/L | 0.16 | 0.09 |
| Concentration of Medium VLDL Particles, mmol/L | 3.46e-5 | 1.21e-5 |
| Total Lipids in Medium VLDL, mmol/L | 0.57 | 0.20 |
| Phospholipids in Medium VLDL, mmol/L | 0.13 | 0.05 |
| Cholesterol in Medium VLDL, mmol/L | 0.17 | 0.06 |
| Cholesteryl Esters in Medium VLDL, mmol/L | 0.09 | 0.04 |
| Free Cholesterol in Medium VLDL, mmol/L | 0.08 | 0.03 |
| Triglycerides in Medium VLDL, mmol/L | 0.27 | 0.11 |
| Concentration of Small VLDL Particles, mmol/L | 3.89e-5 | 1.26e-5 |
| Total Lipids in Small VLDL, mmol/L | 0.41 | 0.13 |
| Phospholipids in Small VLDL, mmol/L | 0.10 | 0.03 |
| Cholesterol in Small VLDL, mmol/L | 0.16 | 0.05 |
| Cholesteryl Esters in Small VLDL, mmol/L | 0.10 | 0.03 |
| Free Cholesterol in Small VLDL, mmol/L | 0.06 | 0.02 |
| Triglycerides in Small VLDL, mmol/L | 0.16 | 0.06 |
| Concentration of Very Small VLDL Particles, mmol/L | 5.49e-5 | 1.35e-5 |
| Total Lipids in Very Small VLDL, mmol/L | 0.36 | 0.09 |
| Phospholipids in Very Small VLDL, mmol/L | 0.10 | 0.03 |
| Cholesterol in Very Small VLDL, mmol/L | 0.19 | 0.05 |
| Cholesteryl Esters in Very Small VLDL, mmol/L | 0.13 | 0.04 |
| Free Cholesterol in Very Small VLDL, mmol/L | 0.06 | 0.01 |
| Triglycerides in Very Small VLDL, mmol/L | 0.07 | 0.02 |
| Concentration of IDL Particles, mmol/L | 5.49e-5 | 7.52e-5 |
| Total Lipids in IDL, mmol/L | 1.23 | 0.28 |
| Phospholipids in IDL, mmol/L | 0.29 | 0.06 |
| Cholesterol in IDL, mmol/L | 0.84 | 0.21 |
| Cholesteryl Esters in IDL, mmol/L | 0.62 | 0.16 |
| Free Cholesterol in IDL, mmol/L | 0.22 | 0.06 |
| Triglycerides in IDL, mmol/L | 0.10 | 0.03 |
| Concentration of Large LDL Particles, mmol/L | 7.30e-4 | 1.73e-4 |
| Total Lipids in Large LDL, mmol/L | 1.57 | 0.37 |
| Phospholipids in Large LDL, mmol/L | 0.36 | 0.08 |
| Cholesterol in Large LDL, mmol/L | 1.12 | 0.28 |
| Cholesteryl Esters in Large LDL, mmol/L | 0.83 | 0.20 |
| Free Cholesterol in Large LDL, mmol/L | 0.29 | 0.08 |
| Triglycerides in Large LDL, mmol/L | 0.10 | 0.02 |
| Concentration of Medium LDL Particles, mmol/L | 0.0003 | 0.0001 |
| Total Lipids in Medium LDL, mmol/L | 0.61 | 0.16 |
| Phospholipids in Medium LDL, mmol/L | 0.16 | 0.04 |
| Cholesterol in Medium LDL, mmol/L | 0.42 | 0.11 |
| Cholesteryl Esters in Medium LDL, mmol/L | 0.30 | 0.09 |
| Free Cholesterol in Medium LDL, mmol/L | 0.12 | 0.03 |
| Triglycerides in Medium LDL, mmol/L | 0.03 | 0.01 |
| Concentration of Small LDL Particles, mmol/L | 0.0002 | 0.00003 |
| Total Lipids in Small LDL, mmol/L | 0.28 | 0.06 |
| Phospholipids in Small LDL, mmol/L | 0.09 | 0.02 |
| Cholesterol in Small LDL, mmol/L | 0.18 | 0.04 |
| Cholesteryl Esters in Small LDL, mmol/L | 0.13 | 0.03 |
| Free Cholesterol in Small LDL, mmol/L | 0.05 | 0.01 |
| Triglycerides in Small LDL, mmol/L | 0.02 | 0.01 |
| Concentration of Very Large HDL Particles, mmol/L | 0.0002 | 0.0001 |
| Total Lipids in Very Large HDL, mmol/L | 0.17 | 0.08 |
| Phospholipids in Very Large HDL, mmol/L | 0.08 | 0.04 |
| Cholesterol in Very Large HDL, mmol/L | 0.08 | 0.03 |
| Cholesteryl Esters in Very Large HDL, mmol/L | 0.06 | 0.03 |
| Free Cholesterol in Very Large HDL, mmol/L | 0.02 | 0.01 |
| Triglycerides in Very Large HDL, mmol/L | 0.007 | 0.003 |
| Concentration of Large HDL Particles, mmol/L | 0.001 | 0.001 |
| Total Lipids in Large HDL, mmol/L | 0.65 | 0.32 |
| Phospholipids in Large HDL, mmol/L | 0.32 | 0.15 |
| Cholesterol in Large HDL, mmol/L | 0.30 | 0.17 |
| Cholesteryl Esters in Large HDL, mmol/L | 0.23 | 0.13 |
| Free Cholesterol in Large HDL, mmol/L | 0.07 | 0.04 |
| Triglycerides in Large HDL, mmol/L | 0.03 | 0.01 |
| Concentration of Medium HDL Particles, mmol/L | 0.004 | 0.001 |
| Total Lipids in Medium HDL, mmol/L | 1.03 | 0.22 |
| Phospholipids in Medium HDL, mmol/L | 0.48 | 0.10 |
| Cholesterol in Medium HDL, mmol/L | 0.49 | 0.12 |
| Cholesteryl Esters in Medium HDL, mmol/L | 0.40 | 0.10 |
| Free Cholesterol in Medium HDL, mmol/L | 0.09 | 0.02 |
| Triglycerides in Medium HDL, mmol/L | 0.05 | 0.02 |
| Concentration of Small HDL Particles, mmol/L | 0.009 | 0.001 |
| Total Lipids in Small HDL, mmol/L | 1.15 | 0.15 |
| Phospholipids in Small HDL, mmol/L | 0.66 | 0.09 |
| Cholesterol in Small HDL, mmol/L | 0.44 | 0.06 |
| Cholesteryl Esters in Small HDL, mmol/L | 0.33 | 0.05 |
| Free Cholesterol in Small HDL, mmol/L | 0.11 | 0.02 |
| Triglycerides in Small HDL, mmol/L | 0.05 | 0.02 |

**Table S3**. Summary of missing data of covariates

| Covariates | Missing (N) | Percentage (%) |
| --- | --- | --- |
| Sex | 0 | 0 |
| Age | 0 | 0 |
| Education | 2348 | 0.79 |
| Employment | 815 | 0.27 |
| Townsend deprivation index | 333 | 0.11 |
| Assessment centre region | 0 | 0 |
| Smoking status | 981 | 0.33 |
| Drinking status | 176 | 0.06 |
| Physical activity | 8953 | 3.01 |
| Body mass index | 217 | 0.07 |
| Sleep duration | 0 | 0 |
| Hypertension | 156 | 0.05 |
| Diabetes | 21 | 0.007 |
| Stroke | 14 | 0.005 |
| Coronary heart disease | 4 | 0.001 |
| Heart failure | 2 | 0.001 |
| Ischemic heart disease | 0 | 0 |
| Lung-related diseases | 4099 | 1.38 |

**Table S4.** Baseline characteristics by lung function (*n* = 280,032)

| **Characteristics** | **Overall** | **Lung function** | | ***P* value** |
| --- | --- | --- | --- | --- |
|  | **(*N*=280032)** | **Normal** | **Impaired** |  |
|  |  | **(*N*=209244)** | **(*N*=70788)** |  |
| Age, mean (SD) | 56.5 (8.0) | 56.0 (8.0) | 58.0 (7.9) | <0.001 |
| Sex, n (%) |  |  |  | <0.001 |
| Female | 147923 (52.8) | 114001 (54.5) | 33922 (47.9) |  |
| Male | 132109 (47.2) | 95243 (45.5) | 36866 (52.1) |  |
| Education, n (%) |  |  |  | <0.001 |
| College or university | 96896 (34.6) | 75344 (36.0) | 21552 (30.5) |  |
| Others | 183136 (65.4) | 133900 (64.0) | 49236 (69.5) |  |
| Employment, n (%) |  |  |  | <0.001 |
| Employed | 171392 (61.2) | 132821 (63.5) | 38571 (54.5) |  |
| Non-employed | 108640 (38.8) | 76423 (36.5) | 32217 (45.5) |  |
| Townsend deprivation index, mean (SD) | -1.6 (2.9) | -1.7 (2.8) | -1.3 (3.0) | <0.001 |
| Assessment center region, n (%) |  |  |  | <0.001 |
| England | 218065 (77.9) | 163546 (78.2) | 54519 (77.0) |  |
| Scotland | 29409 (10.5) | 22220 (10.6) | 7189 (10.2) |  |
| Wales | 32558 (11.6) | 23478 (11.2) | 9080 (12.8) |  |
| Smoking status, n (%) |  |  |  | <0.001 |
| Never | 157149 (56.1) | 122904 (58.7) | 34245 (48.4) |  |
| Previous | 100561 (35.9) | 73006 (34.9) | 27555 (38.9) |  |
| Current | 22322 (8.0) | 13334 (6.4) | 8988 (12.7) |  |
| Drinking status, n (%) |  |  |  | <0.001 |
| Never | 7906 (2.8) | 5702 (2.7) | 2204 (3.1) |  |
| Previous | 7446 (2.7) | 5159 (2.5) | 2287 (3.2) |  |
| Current | 264680 (94.5) | 198383 (94.8) | 66297 (93.7) |  |
| Physical activity, n (%) |  |  |  | <0.001 |
| Physical active | 146902 (52.5) | 111575 (53.3) | 35327 (49.9) |  |
| Physical inactive | 133130 (47.5) | 97669 (46.7) | 35461 (50.1) |  |
| BMI, mean (SD) | 27.2 (4.6) | 27.1 (4.4) | 27.6 (5.0) | <0.001 |
| Sleep duration (%) |  |  |  |  |
| Normal (7-8 h) | 196959 (70.3) | 148767 (71.1) | 48192 (68.1) | <0.001 |
| Short (< 7 h) | 65006 (23.2) | 47786 (22.8) | 17220 (24.3) |  |
| Long (> 8 h) | 18067 (6.5) | 12691 (6.1) | 5376 (7.6) |  |
| Hypertension, n (%) | 69215 (24.7) | 48068 (23.0) | 21147 (29.9) | <0.001 |
| Diabetes, n (%) | 11483 (4.1) | 7241 (3.5) | 4242 (6.0) | <0.001 |
| Stroke, n (%) | 4814 (1.7) | 3114 (1.5) | 1700 (2.4) | <0.001 |
| Coronary heart disease, n (%) | 12100 (4.3) | 7394 (3.5) | 4706 (6.7) | <0.001 |
| Heart failure, n (%) | 1063 (0.4) | 506 (0.2) | 557 (0.8) | <0.001 |
| Ischemic heart disease, n (%) | 7133 (2.6) | 4275 (2.0) | 2858 (4.0) | <0.001 |
| Lung-related diseases, n (%) | 35501 (12.7) | 20210 (9.7) | 15291 (21.6) | <0.001 |
| Incident depression, n (%) | 9514(3.4) | 6635(3.2) | 2879(4.1) | <0.001 |
| FEV_1_% predicted, mean (SD) | 93.4 (16.4) | 99.4 (12.0) | 75.9 (14.9) | <0.001 |
| FVC % predicted, mean (SD) | 97.1 (15.2) | 100.7 (12.1) | 86.7 (18.3) | <0.001 |

Abbreviations: BMI, body mass index; SD, standard deviation; FEV_1_, forced expiratory volume in 1s; FVC, forced vital capacity.

# Table S5. Sensitivity analyses for association between incident depression and lung function with further adjustment for covariates

| **Variables** | **Risk of incident depression** | | | | | |
| --- | --- | --- | --- | --- | --- | --- |
|  | **Model 3 + cumulative cigarette consumption (*n*=280,032)** | | **Model 3 + cumulative cigarette consumption +** **duration of passive smoking (*n*=261,511)** | | **Model 3 +** **PM_2.5_ + NO_2_ (*n*=280,032)** | |
|  | **HR (95% CI)** | ***P* value** | **HR (95% CI)** | ***P* value** | **HR (95% CI)** | ***P* value** |
| **FVC(% Predicted)** |  |  |  |  |  |  |
| Quartile 1 (lowest) | 1(Reference) |  | 1(Reference) |  | 1(Reference) |  |
| Quartile 2 | 0.878(0.831-0.928) | <0.001 | 0.881(0.830-0.935) | <0.001 | 0.872(0.825-0.921) | <0.001 |
| Quartile 3 | 0.893(0.844-0.945) | <0.001 | 0.902(0.849-0.959) | <0.001 | 0.884(0.835-0.935) | <0.001 |
| Quartile 4 (highest) | 0.887(0.836-0.940) | <0.001 | 0.887(0.834-0.944) | <0.001 | 0.873(0.824-0.926) | <0.001 |
| **FEV_1_(% Predicted)** |  |  |  |  |  |  |
| Quartile 1 (lowest) | 1(Reference) |  | 1(Reference) |  | 1(Reference) |  |
| Quartile 2 | 0.914(0.865-0.966) | <0.001 | 0.915(0.863-0.971) | <0.001 | 0.904(0.856-0.955) | <0.001 |
| Quartile 3 | 0.875(0.826-0.926) | <0.001 | 0.878(0.826-0.933) | <0.001 | 0.861(0.813-0.912) | <0.001 |
| Quartile 4 (highest) | 0.872(0.822-0.925) | <0.001 | 0.870(0.817-0.926) | <0.001 | 0.854(0.805-0.906) | <0.001 |
| Lung function |  |  |  |  |  |  |
| Normal | 1(Reference) |  | 1(Reference) |  | 1(Reference) |  |
| Impaired | 1.106(1.056-1.158) | <0.001 | 1.104(1.050-1.160) | <0.001 | 1.123(1.073-1.176) | <0.001 |

Abbreviations: HR, hazards ratio; CI, confidence interval; FEV_1_, forced expiratory volume in 1 second; FVC, forced vital capacity. PM_2.5_, particulate matter with a diameter of 2.5 micrometers or less; NO_2_, nitrogen dioxide. Pack-years was calculated as a cumulative exposure measure of smoking burden. One pack-year is defined as 20 cigarettes smoked every day for 1 year. Model 3 adjusted for age at baseline, sex, education, employment, assessment center, Townsend Deprivation Index, height, smoking status, drinking status, physical activity, BMI, sleep duration, hypertension, diabetes, heart failure, stroke, coronary heart disease, ischemic heart disease, and pre-existing lung diseases.

# Table S6. Sensitivity analyses for association between incident depression and lung function with different exclusion criteria

| **Variables** | **Risk of incident depression** | | | |
| --- | --- | --- | --- | --- |
|  | **Model 3^a^ + after excluding lung-related diseases at baseline (*n*=244,531)** | | **Model 3^b^ + after excluding depression cases occurring in the first two years of follow-up (*n*=277,915)** | |
|  | **HR (95% CI)** | ***P* value** | **HR (95% CI)** | ***P* value** |
| **FVC(% Predicted)** |  |  |  |  |
| Quartile 1 (lowest) | 1(Reference) |  | 1(Reference) |  |
| Quartile 2 | 0.884(0.831-0.941) | <0.001 | 0.875(0.825-0.928) | <0.001 |
| Quartile 3 | 0.901(0.846-0.959) | 0.001 | 0.886(0.834-0.941) | <0.001 |
| Quartile 4 (highest) | 0.906(0.850-0.966) | 0.003 | 0.876(0.823-0.932) | <0.001 |
| **FEV_1_(% Predicted)** |  |  |  |  |
| Quartile 1 (lowest) | 1(Reference) |  | 1(Reference) |  |
| Quartile 2 | 0.916(0.861-0.974) | 0.005 | 0.896(0.845-0.950) | <0.001 |
| Quartile 3 | 0.878(0.825-0.936) | <0.001 | 0.854(0.804-0.908) | <0.001 |
| Quartile 4 (highest) | 0.868(0.814-0.926) | <0.001 | 0.849(0.797-0.903) | <0.001 |
| Lung function |  |  |  |  |
| Normal | 1(Reference) |  | 1(Reference) |  |
| Impaired | 1.110(1.054-1.169) | <0.001 | 1.136(1.082-1.192) | <0.001 |

Abbreviations: HR, hazards ratio; CI, confidence interval; FEV_1_, forced expiratory volume in 1 second; FVC, forced vital capacity.

Lung-related diseases included asthma, chronic obstructive airways disease/COPD, emphysema/chronic bronchitis, and other respiratory problems.

^a^ adjusted for age at baseline, sex, education, employment, assessment center, Townsend Deprivation Index, height, smoking status, drinking status, physical activity, BMI, sleep duration, hypertension, diabetes, heart failure, stroke, coronary heart disease, and ischemic heart disease.

^b^ adjusted for age at baseline, sex, education, employment, assessment center, Townsend Deprivation Index, height, smoking status, drinking status, physical activity, BMI, sleep duration, hypertension, diabetes, heart failure, stroke, coronary heart disease, ischemic heart disease, and lung-related diseases.

# Table S7. Sensitivity analyses for association between lung function and risk of incident depression with multiple imputation for missing covariates (*n* = 297,037)

| **Variables** | **Risk of incident depression** | | | | | |
| --- | --- | --- | --- | --- | --- | --- |
|  | **Model 1** | | **Model 2** | | **Model 3** | |
|  | **HR (95% CI)** | ***P* value** | **HR (95% CI)** | ***P* value** | **HR (95% CI)** | ***P* value** |
| **FVC(% Predicted)** |  |  |  |  |  |  |
| Quartile 1 (lowest) | **Ref** |  | **Ref** |  | **Ref** |  |
| Quartile 2 | 0.793(0.752-0.836) | <0.001 | 0.852(0.808-0.898) | <0.001 | 0.884(0.838-0.933) | <0.001 |
| Quartile 3 | 0.758(0.719-0.800) | <0.001 | 0.847(0.802-0.894) | <0.001 | 0.891(0.843-0.941) | <0.001 |
| Quartile 4 (highest) | 0.717(0.679-0.757) | <0.001 | 0.830(0.785-0.878) | <0.001 | 0.883(0.835-0.934) | <0.001 |
| **FEV_1_(% Predicted)** |  |  |  |  |  |  |
| Quartile 1 (lowest) | **Ref** |  | **Ref** |  | **Ref** |  |
| Quartile 2 | 0.810(0.769-0.853) | <0.001 | 0.861(0.820-0.911) | <0.001 | 0.906(0.859-0.955) | 0.001 |
| Quartile 3 | 0.737(0.699-0.778) | <0.001 | 0.810(0.768-0.856) | <0.001 | 0.864(0.817-0.912) | <0.001 |
| Quartile 4 (highest) | 0.701(0.663-0.740) | <0.001 | 0.790(0.747-0.836) | <0.001 | 0.855(0.808-0.905) | <0.001 |
| Lung function |  |  |  |  |  |  |
| Normal | **Ref** |  | **Ref** |  | **Ref** |  |
| Impaired | 1.279(1.226-1.335) | <0.001 | 1.189(1.139-1.242) | <0.001 | 1.120(1.072-1.170) | <0.001 |

Abbreviations: HR, hazards ratio; CI, confidence interval; FEV_1_, forced expiratory volume in 1 second; FVC, forced vital capacity.

Model 1 adjusted for age at baseline, sex, education, employment, assessment center, Townsend Deprivation Index, and height.

Model 2 adjusted for model 1 plus smoking status, drinking status, physical activity, BMI, sleep duration.

Model 3 adjusted for model 2 plus hypertension, diabetes, heart failure, stroke, coronary heart disease, ischemic heart disease,

and pre-existing lung diseases (yes or no).

# Table S8. Sensitivity analyses for association between lung function and risk of incident depression with further excluding prevalent depression as measured by the PHQ-2 scale at baseline (*n* = 271,122)

| **Variables** | **Risk of incident depression** | | | | | |
| --- | --- | --- | --- | --- | --- | --- |
|  | **Model 1** | | **Model 2** | | **Model 3** | |
|  | **HR (95% CI)** | ***P* value** | **HR (95% CI)** | ***P* value** | **HR (95% CI)** | ***P* value** |
| **FVC(% Predicted)** |  |  |  |  |  |  |
| Quartile 1 (lowest) | 1(Reference) |  | 1(Reference) |  | 1(Reference) |  |
| Quartile 2 | 0.792 (0.747-0.839) | <0.001 | 0.848 (0.800-0.899) | <0.001 | 0.879 (0.829-0.932) | <0.001 |
| Quartile 3 | 0.763 (0.719-0.809) | <0.001 | 0.848 (0.799-0.900) | <0.001 | 0.890 (0.838-0.945) | <0.001 |
| Quartile 4 (highest) | 0.707 (0.666-0.751) | <0.001 | 0.815 (0.766-0.867) | <0.001 | 0.866 (0.814-0.921) | <0.001 |
| **FEV_1_(% Predicted)** |  |  |  |  |  |  |
| Quartile 1 (lowest) | 1(Reference) |  | 1(Reference) |  | 1(Reference) |  |
| Quartile 2 | 0.798 (0.753-0.845) | <0.001 | 0.852 (0.805-0.903) | <0.001 | 0.894 (0.844-0.947) | <0.001 |
| Quartile 3 | 0.724 (0.683-0.768) | <0.001 | 0.798 (0.752-0.847) | <0.001 | 0.850 (0.800-0.903) | <0.001 |
| Quartile 4 (highest) | 0.691 (0.651-0.734) | <0.001 | 0.785 (0.738-0.834) | <0.001 | 0.849 (0.798-0.903) | <0.001 |
| **Lung function** |  |  |  |  |  |  |
| Normal | 1(Reference) |  | 1(Reference) |  | 1(Reference) |  |
| Impaired | 1.299(1.239-1.361) | <0.001 | 1.211 (1.155-1.270) | <0.001 | 1.139 (1.085-1.195) | <0.001 |

Note, HR, hazards ratio; CI, confidence interval; FEV_1_, forced expiratory volume in 1 second; FVC, forced vital capacity. Model 1 adjusted for age at baseline, sex, education, employment, assessment center, Townsend Deprivation Index, and height. Model 2 adjusted for model 1 plus smoking status, drinking status, physical activity, BMI, sleep duration. Model 3 adjusted for model 2 plus hypertension, diabetes, heart failure, stroke, coronary heart disease, ischemic heart disease, and pre-existing lung diseases (yes or no).

**Table S9.** Selection of biomarkers as potential mediators between lung function and incident depression (*n* = 231,193)

|  | Associations between lung function and biomarkers | | Associations between biomarkers and incident depression | |
| --- | --- | --- | --- | --- |
| Blood Biomarkers | *Beta*^a^ (95% *CI*) | *FDR* | *HR*^b^ (95% CI) | *FDR* |
| Inflammatory-related biomarkers |  |  |  |  |
| **Leukocyte count, 10^9 cells/L** | 0.1143 (0.1049, 0.1237) | < 2E-16 | 1.0240 (1.0151-1.0329) | 4.16E-7 |
| **Neutrophil count, 10^9 cells/L** | 0.1315 (0.1221, 0.1408) | < 2E-16 | 1.0554 (1.0340-1.0773) | 9.58E-7 |
| **Neutrophil percentage, %** | 0.0426 (0.0330, 0.0522) | < 2E-16 | 1.0358 (1.0126-1.0595) | 0.0037 |
| Monocyte count, 10^9 cells/L | 0.0547 (0.0448, 0.0645) | < 2E-16 | 1.0049 (0.9905-1.0195) | 0.5872 |
| Monocyte percentage, % | -0.0004 (-0.0097, 0.0089) | 0.9350 | 0.9769 (0.9528-1.0015) | 0.0842 |
| **Lymphocyte count, 10^9 cells/L** | 0.0237 (0.0142, 0.0332) | 2.50E-06 | 1.0171 (1.0031-1.0313) | 0.0221 |
| **Lymphocyte percentage, %** | -0.0716 (-0.0811, -0.0621) | < 2E-16 | 0.9709 (0.9489-0.9933) | 0.0162 |
| **C reactive protein, mg/L** | 0.1266 (0.1174, 0.1359) | < 2E-16 | 1.0316 (1.0114-1.0522) | 0.0033 |
| **Platelet count, 10^9 cells/L** | 0.0361 (0.0268, 0.0454) | 3.13E-13 | 1.0470 (1.0241-1.0705) | < 0.0001 |
| Erythrocyte-related biomarkers |  |  |  |  |
| **Erythrocyte count, 10^12 cells/L** | -0.0092 (-0.0172, -0.0011) | 0.0393 | 0.8854 (0.8625-0.9089) | < 2E-16 |
| High light scatter reticulocyte count, 10^12 cells/L | 0.0459 (0.0371, 0.0548) | < 2E-16 | 1.0015 (0.9784-1.0251) | 0.9422 |
| Reticulocyte count, 10^12 cells/L | 0.0239 (0.0146, 0.0330) | 1.60E-06 | 0.9944 (0.9702-1.0191) | 0.7148 |
| **Red blood cell distribution width, %** | 0.0751 (0.0655, 0.0846) | < 2E-16 | 1.0406 (1.0202-1.0614) | 0.0002 |
| Haematocrit percentage, % | -0.0011 (-0.0089, 0.0066) | 0.8503 | 0.9084 (0.8842-0.9332) | 2.25E-11 |
| **Haemoglobin concentration, g/dL** | -0.0112 (-0.0187, -0.0037) | 0.0069 | 0.8982 (0.8739-0.9232) | 2.32E-13 |
| Renal function-related biomarkers |  |  |  |  |
| **Cystatin C, mg/L** | 0.1097(0.1012, 0.1181) | < 2E-16 | 1.0336 (1.0124-1.0552) | 0.0032 |
| **Urate, μmol/L** | 0.0358 (0.0284, 0.0433) | < 2E-16 | 0.9471 (0.9207-0.9743) | 0.0004 |
| Urea, mmol/L | 0.0035 (-5.7e-03, 0.0126) | 0.5462 | 0.9998 (0.9771-1.0231) | 0.9884 |
| Liver function-related biomarkers |  |  |  |  |
| Alanine aminotransferase, U/L | -0.0056 (-0.0146, 0.0033) | 0.2899 | 1.0152 (0.9929-1.0379) | 0.2209 |
| **Alkaline phosphatase, U/L** | 0.0635 (0.0544, 0.0727) | < 2E-16 | 1.0351 (1.0137-1.0569) | 0.0023 |
| **Aspartate aminotransferase, U/L** | 0.0104 (0.0012, 0.0195) | 0.0393 | 1.0464 (1.0279-1.0652) | 2.05E-06 |
| **Gamma-glutamyl transferase, U/L** | 0.0336 (0.0243, 0.0430) | 1.16E-11 | 1.0661 (1.0488-1.0837) | 2.32E-13 |
| **Total bilirubin, μmol/L** | -0.0450 (-0.0542, -0.0357) | < 2E-16 | 0.9481 (0.9237-0.9732) | 1.59E-04 |
| **Total protein, g/L** | -0.0246 (-0.0341, -0.0150) | 1.60E-06 | 0.9508 (0.9295-0.9725) | 3.48E-05 |
| **Albumin, g/L** | -0.1173 (-0.1266, -0.1079) | < 2E-16 | 0.9323 (0.9111-0.9540) | 1.28E-08 |

Lung function was dichotomous and the normal level was considered as the reference group. Biomarkers concentrations were standardized by z-score.

^a^Models were adjusted for age, sex, education, employment, assessment center, Townsend deprivation index, height, smoking status, drinking status, physical activity, BMI, sleep duration, hypertension, diabetes, heart failure, stroke, coronary heart disease, ischemic heart disease, and lung-related diseases.

^b^Models were adjusted for age, sex, education, employment, assessment center, Townsend deprivation index, height, smoking status, drinking status, physical

activity, BMI, sleep duration, hypertension, diabetes, heart failure, stroke, coronary heart disease, ischemic heart disease, lung-related diseases, and lung function.

Bolded indicated that biomarkers may be potential mediators in the association between lung function and the risk of depression. FDR, false discovery rate; PM, proportion mediated; CI, confidence interval; HR, hazard ratio.

**Table S10**. Selection of metabolites as potential mediators between lung function and incident depression (*n* = 62,488)

| Metabolites | Associations between lung function and metabolites | | Associations between metabolites and incident depression | |
| --- | --- | --- | --- | --- |
|  | Beta^a^ (95% CI) | *FDR* | HR^b^ (95% CI) | *FDR* |
| Total Cholesterol, mmol/L | -0.0490 (-0.0657, -0.0323) | 6.63E-08 | 0.9784 (0.9364-1.0224) | 0.3311 |
| Total Cholesterol Minus HDL-C, mmol/L | -0.0525 (-0.0696, -0.0353) | 2.26E-08 | 0.9724 (0.9327-1.0159) | 0.1998 |
| Remnant Cholesterol, mmol/L | -0.0430 (-0.0601, -0.0259) | 3.38E-06 | 0.9804 (0.9394-1.0232) | 0.3639 |
| VLDL Cholesterol, mmol/L | -0.0324 (-0.0496, -0.0151) | 0.0007 | 0.9830 (0.9422-1.0265) | 0.4268 |
| Clinical LDL Cholesterol, mmol/L | -0.0607 (-0.0779, -0.0436) | 1.65E-10 | 0.9687 (0.9265-1.0128) | 0.1611 |
| LDL Cholesterol, mmol/L | -0.0597 (-0.0770, -0.0424) | 3.87E-10 | 0.9660 (0.9256-1.0081) | 0.1115 |
| HDL Cholesterol, mmol/L | -0.0060 (-0.0212, 0.0092) | 0.5365 | 1.0163 (0.9685-1.0665) | 0.5094 |
| Total Triglycerides, mmol/L | 0.0093 (-0.0075, 0.0261) | 0.3584 | 1.0150 (0.9732-1.0585) | 0.4883 |
| Triglycerides in VLDL, mmol/L | 0.0059 (-0.0108, 0.0225) | 0.5799 | 1.0112 (0.9692-1.0551) | 0.6065 |
| Triglycerides in LDL, mmol/L | 0.0201 (0.0028, 0.0374) | 0.0399 | 1.0193 (0.9786-1.0617) | 0.3580 |
| Triglycerides in HDL, mmol/L | 0.0224 (0.0049, 0.0400) | 0.0235 | 1.0335 (0.9928-1.0759) | 0.1075 |
| Total Phospholipids in Lipoprotein Particles, mmol/L | -0.0206 (-0.0373, -0.0039) | 0.0286 | 1.0073 (0.9646-1.0520) | 0.7408 |
| Phospholipids in VLDL, mmol/L | -0.0155 (-0.0325, 0.0016) | 0.1167 | 0.9946 (0.9534-1.0376) | 0.8031 |
| Phospholipids in LDL, mmol/L | -0.0559 (-0.0732, -0.0387) | 3.67E-09 | 0.9663 (0.9261-1.0083) | 0.1146 |
| Phospholipids in HDL, mmol/L | 0.0109 (-0.0050, 0.0267) | 0.2463 | 1.0366 (0.9904-1.0849) | 0.1226 |
| Total Esterified Cholesterol, mmol/L | -0.0494 (-0.0660, -0.0328) | 4.51E-08 | 0.9783 (0.9360-1.0225) | 0.3304 |
| Cholesteryl Esters in VLDL, mmol/L | -0.0376 (-0.0548, -0.0203) | 6.28E-05 | 0.9791 (0.9384-1.0216) | 0.3303 |
| Cholesteryl Esters in LDL, mmol/L | -0.0573 (-0.0746, -0.0399) | 1.87E-09 | 0.9681 (0.9279-1.0101) | 0.1351 |
| Cholesteryl Esters in HDL, mmol/L | -0.0084 (-0.0237, 0.0068) | 0.3584 | 1.0118 (0.9643-1.0617) | 0.6312 |
| Total Free Cholesterol, mmol/L | -0.0470 (-0.0639, -0.0301) | 2.85E-07 | 0.9792 (0.9376-1.0227) | 0.3425 |
| Free Cholesterol in VLDL, mmol/L | -0.0243 (-0.0414, -0.0072) | 0.0126 | 0.9886 (0.9476-1.0314) | 0.5952 |
| Free Cholesterol in LDL, mmol/L | -0.0641 (-0.0812, -0.0470) | 1.60E-11 | 0.9663 (0.9239-1.0105) | 0.1330 |
| Free Cholesterol in HDL, mmol/L | 0.0027 (-0.0125, 0.0180) | 0.7829 | 1.0312 (0.9830-1.0817) | 0.2090 |
| Total Lipids in Lipoprotein Particles, mmol/L | -0.0306(-0.0478, -0.0134) | 0.0014 | 0.9957 (0.9547-1.0385) | 0.8410 |
| Total Lipids in VLDL, mmol/L | -0.0093 (-0.0262, 0.0077) | 0.3597 | 1.0001 (0.9586-1.0434) | 0.9977 |
| Total Lipids in LDL, mmol/L | -0.0554 (-0.0727, -0.0381) | 4.54E-09 | 0.9687 (0.9284-1.0108) | 0.1427 |
| Total Lipids in HDL, mmol/L | 0.0040 (-0.0116, 0.0196) | 0.6831 | 1.0299 (0.9831-1.0790) | 0.2145 |
| Total Concentration of Lipoprotein Particles, mmol/L | -0.0265 (-0.0431, -0.0099) | 0.0044 | 0.9987 (0.9558-1.0435) | 0.9547 |
| Concentration of VLDL Particles, mmol/L | -0.0239 (-0.0412, -0.0067) | 0.0150 | 0.9912 (0.9504-1.0338) | 0.6812 |
| Concentration of LDL Particles, mmol/L | -0.0517 (-0.0690, -0.0343) | 4.05E-08 | 0.9679 (0.9276-1.0100) | 0.1328 |
| Concentration of HDL Particles, mmol/L | -0.0199(-0.0365, -0.0033) | 0.0337 | 1.0036 (0.9605-1.0485) | 0.8738 |
| Average Diameter for VLDL Particles, nm | -0.0055 (-0.0213, 0.0104) | 0.5823 | 0.9810 (0.9370-1.0270) | 0.4115 |
| Average Diameter for LDL Particles, nm | -0.0183 (-0.0354, -0.0012) | 0.0586 | 0.9702 (0.9298-1.0123) | 0.1629 |
| Average Diameter for HDL Particles, nm | 0.0233 (0.0085, 0.0381) | 0.0051 | 1.0358 (0.9833-1.0910) | 0.1848 |
| Phosphoglycerides, mmol/L | -0.0121 (-0.0289, 0.0046) | 0.2206 | 1.0207 (0.9778-1.0655) | 0.3492 |
| Total Cholines, mmol/L | -0.0192 (-0.0357, -0.0027) | 0.0399 | 1.0138 (0.9704-1.0592) | 0.5380 |
| Phosphatidylcholines, mmol/L | -0.0161 (-0.0326, 0.0004) | 0.0883 | 1.0230 (0.9794-1.0685) | 0.3061 |
| Sphingomyelins, mmol/L | -0.0224 (-0.0386, -0.0062) | 0.0153 | 0.9941 (0.9501-1.0400) | 0.7960 |
| Apolipoprotein B, g/l | -0.0484 (-0.0656, -0.0312) | 2.40E-07 | 0.9721 (0.9316-1.0143) | 0.1923 |
| Apolipoprotein A1, g/l | -0.0026 (-0.0186, 0.0135) | 0.7938 | 1.0219 (0.9768-1.0691) | 0.3474 |
| Total Fatty Acids, mmol/L | -0.0014(-0.0188, 0.0160) | 0.8912 | 1.0138 (0.9732-1.0561) | 0.5123 |
| Degree of Unsaturation, degree | -0.0859 (-0.1023, -0.0694) | < 2E-16 | 0.9687 (0.9260-1.0133) | 0.1657 |
| Omega-3 Fatty Acids, mmol/L | -0.0624 (-0.0797, -0.0450) | 9.84E-11 | 0.9962 (0.9544-1.0398) | 0.8615 |
| Omega-6 Fatty Acids, mmol/L | -0.0354 (-0.0526, -0.0183) | 0.0002 | 0.9882 (0.9470-1.0311) | 0.5835 |
| Polyunsaturated Fatty Acids, mmol/L | -0.0472 (-0.0644, -0.0300) | 4.14E-07 | 0.9889 (0.9477-1.0319) | 0.6082 |
| Monounsaturated Fatty Acids, mmol/L | 0.0272 (0.0102, 0.0444) | 0.0045 | 1.0206 (0.9797-1.0632) | 0.3295 |
| Saturated Fatty Acids, mmol/L | 0.0131 (-0.0043, 0.0305) | 0.2005 | 1.0259 (0.9853-1.0682) | 0.2152 |
| Linoleic Acid, mmol/L | -0.0401 (-0.0574, -0.0228) | 2.06E-05 | 0.9807 (0.9398-1.0234) | 0.3698 |
| Docosahexaenoic Acid, mmol/L | -0.0744 (-0.0913, -0.0574) | < 2E-16 | 0.9827 (0.9397-1.0277) | 0.4441 |
| Alanine, mmol/L | -0.0251 (-0.0428, -0.0074) | 0.0126 | 0.9668 (0.9259-1.0096) | 0.1263 |
| Glutamine, mmol/L | -0.0060 (-0.0241, 0.0121) | 0.5954 | 1.0644 (1.0196-1.1111) | 0.0045 |
| Glycine, mmol/L | -0.0025 (-0.0195, 0.0145) | 0.8073 | 0.9814 (0.9393-1.0254) | 0.4026 |
| Histidine, mmol/L | -0.0481(-0.0664, -0.0297) | 1.27E-06 | 1.0252 (0.9864-1.0656) | 0.2062 |
| Total Concentration of Branched-Chain Amino Acids, mmol/L | 0.0079 (-0.0095, 0.0253) | 0.4642 | 0.9905 (0.9487-1.0342) | 0.6657 |
| Isoleucine, mmol/L | 0.0188 (0.0009, 0.0367) | 0.0635 | 1.0220 (0.9806-1.0651) | 0.3023 |
| Leucine, mmol/L | 0.0049 (-0.0124, 0.0224) | 0.6536 | 0.9892 (0.9473-1.0330) | 0.6231 |
| Valine, mmol/L | 0.0047 (-0.0127, 0.0221) | 0.6656 | 0.9776 (0.9362-1.0209) | 0.3065 |
| Phenylalanine, mmol/L | 0.0528 (0.0344, 0.0711) | 1.20E-07 | 1.0179 (0.9776-1.0598) | 0.3904 |
| Tyrosine, mmol/L | 0.0399 (0.0218, 0.0580) | 4.98E-05 | 0.9960 (0.9554-1.0384) | 0.8502 |
| Glucose, mmol/L | -0.0083 (-0.0251, 0.0084) | 0.4117 | 1.0272 (0.9879-1.0680) | 0.1775 |
| Lactate, mmol/L | 0.0138 (-0.0043, 0.0320) | 0.1954 | 0.9560 (0.9152-0.9986) | 0.0431 |
| Pyruvate, mmol/L | 0.0168 (0.0013, 0.0323) | 0.0563 | 0.9656 (0.9168-1.0169) | 0.1850 |
| Citrate, mmol/L | -0.0309 (-0.0488, -0.0130) | 0.0020 | 0.9782 (0.9374-1.0207) | 0.6109 |
| 3-Hydroxybutyrate, mmol/L | 0.0021 (-0.0161, 0.0202) | 0.8489 | 0.9770 (0.9345-1.0214) | 0.6109 |
| Acetate, mmol/L | -0.0114 (-0.0183, -0.0045) | 0.0034 | 0.9842 (0.8688-1.1149) | 0.9139 |
| Acetoacetate, mmol/L | 0.0239 (0.0056, 0.0421) | 0.0211 | 1.0082 (0.9689-1.0490) | 0.8577 |
| Acetone, mmol/L | 0.0022 (-0.0154, 0.0198) | 0.8411 | 0.9980 (0.9578-1.0398) | 0.9776 |
| Creatinine, mmol/L | -0.0109 (-0.0257, 0.0039) | 0.2158 | 0.9817 (0.9331-1.0329) | 0.6926 |
| Albumin, g/l | -0.1126 (-0.1306, -0.0947) | < 2E-16 | 0.9627 (0.9214-1.0059) | 0.6109 |
| Glycoprotein Acetyls, mmol/L | 0.0866 (0.0696, 0.1035) | < 2E-16 | 1.0077 (0.9648-1.0525) | 0.8682 |
| Concentration of Chylomicrons and Extremely Large VLDL Particles, mmol/L | 0.0226 (0.0056, 0.0397) | 0.0200 | 1.0205 (0.9784-1.0645) | 0.6408 |
| Total Lipids in Chylomicrons and Extremely Large VLDL, mmol/L | 0.0226 (0.0055, 0.0396) | 0.0200 | 1.0226 (0.9805-1.0665) | 0.6109 |
| Phospholipids in Chylomicrons and Extremely Large VLDL, mmol/L | 0.0216 (0.0046, 0.0386) | 0.0248 | 1.0165 (0.9742-1.0606) | 0.6916 |
| Cholesterol in Chylomicrons and Extremely Large VLDL, mmol/L | 0.0145 (-0.0026, 0.0315) | 0.1468 | 1.0079 (0.9656-1.0520) | 0.8661 |
| Cholesteryl Esters in Chylomicrons and Extremely Large VLDL, mmol/L | 0.0110 (-0.0613, 0.0280) | 0.2784 | 1.0026 (0.9602-1.0467) | 0.9776 |
| Free Cholesterol in Chylomicrons and Extremely Large VLDL, mmol/L | 0.0185 (0.0015, 0.0356) | 0.0562 | 1.0141 (0.9720-1.0581) | 0.7119 |
| Triglycerides in Chylomicrons and Extremely Large VLDL, mmol/L | 0.0251(0.0080, 0.0422) | 0.0094 | 1.0282 (0.9864-1.0718) | 0.6109 |
| Concentration of Very Large VLDL Particles, mmol/L | 0.0046 (-0.0124, 0.0216) | 0.6657 | 1.0089 (0.9663-1.0534) | 0.8577 |
| Total Lipids in Very Large VLDL, mmol/L | 0.0062 (-0.0108, 0.0232) | 0.5669 | 1.0098 (0.9673-1.0542) | 0.8483 |
| Phospholipids in Very Large VLDL, mmol/L | 0.0031 (-0.0140, 0.0201) | 0.7829 | 1.0070 (0.9645-1.0514) | 0.8856 |
| Cholesterol in Very Large VLDL, mmol/L | -0.0123 (-0.0295, 0.0049) | 0.2253 | 0.9918 (0.9497-1.0357) | 0.8647 |
| Cholesteryl Esters in Very Large VLDL, mmol/L | -0.0230 (-0.0403, -0.0056) | 0.0200 | 0.9809 (0.9393-1.0245) | 0.6621 |
| Free Cholesterol in Very Large VLDL, mmol/L | -0.0011 (-0.0182, 0.0160) | 0.9065 | 1.0032 (0.9609-1.0474) | 0.9655 |
| Triglycerides in Very Large VLDL, mmol/L | 0.0136 (-0.0034, 0.0305) | 0.1751 | 1.0168 (0.9742-1.0612) | 0.6916 |
| Concentration of Large VLDL Particles, mmol/L | -0.0057 (-0.0229, 0.0114) | 0.5947 | 0.9997 (0.9575-1.0439) | 0.9955 |
| Total Lipids in Large VLDL, mmol/L | -0.0078 (-0.0250, 0.0094) | 0.4642 | 0.9988 (0.9567-1.0428) | 0.9852 |
| Phospholipids in Large VLDL, mmol/L | -0.0041 (-0.0211, 0.0130) | 0.7037 | 1.0001 (0.9576-1.0445) | 0.9955 |
| Cholesterol in Large VLDL, mmol/L | -0.0199 (-0.0373, -0.0026) | 0.0420 | 0.9871 (0.9454-1.0307) | 0.7569 |
| Cholesteryl Esters in Large VLDL, mmol/L | -0.0289 (-0.0464, -0.0113) | 0.0034 | 0.9802 (0.9391-1.0232) | 0.6565 |
| Free Cholesterol in Large VLDL, mmol/L | -0.0110 (-0.0281, 0.0061) | 0.2784 | 0.9942 (0.9518-1.0385) | 0.9139 |
| Triglycerides in Large VLDL, mmol/L | -0.0029 (-0.0201, 0.0144) | 0.7917 | 1.0044 (0.9623-1.0483) | 0.9399 |
| Concentration of Medium VLDL Particles, mmol/L | -0.0415 (-0.0593, -0.0237) | 1.82E-05 | 0.9739 (0.9335-1.0161) | 0.6109 |
| Total Lipids in Medium VLDL, mmol/L | -0.0337 (-0.0515, -0.0160) | 0.0006 | 0.9784 (0.9378-1.0207) | 0.6109 |
| Phospholipids in Medium VLDL, mmol/L | -0.0395 (-0.0573, -0.0217) | 4.59E-05 | 0.9753 (0.9348-1.0175) | 0.6109 |
| Cholesterol in Medium VLDL, mmol/L | -0.0533 (-0.0710, -0.0355) | 3.51E-08 | 0.9696 (0.9274-1.0138) | 0.6109 |
| Cholesteryl Esters in Medium VLDL, mmol/L | -0.0565 (-0.0741, -0.0390) | 3.83E-09 | 0.9671 (0.9246-1.0117) | 0.6109 |
| Free Cholesterol in Medium VLDL, mmol/L | -0.0455 (-0.0633, -0.0277) | 2.52E-06 | 0.9710 (0.9306-1.0132) | 0.6109 |
| Triglycerides in Medium VLDL, mmol/L | -0.0135 (-0.0309, 0.0040) | 0.1921 | 0.9917 (0.9503-1.0350) | 0.8647 |
| Concentration of Small VLDL Particles, mmol/L | -0.0210 (-0.0387, -0.0033) | 0.0355 | 0.9846 (0.9438-1.0272) | 0.6926 |
| Total Lipids in Small VLDL, mmol/L | -0.0220 (-0.0397, -0.0043) | 0.0280 | 0.9848 (0.9440-1.0274) | 0.6926 |
| Phospholipids in Small VLDL, mmol/L | -0.0358 (-0.0536, -0.0179) | 0.0002 | 0.9746 (0.9343-1.0168) | 0.6109 |
| Cholesterol in Small VLDL, mmol/L | -0.0382 (-0.0560, -0.0203) | 8.39E-05 | 0.9714 (0.9310-1.0136) | 0.6109 |
| Cholesteryl Esters in Small VLDL, mmol/L | -0.0335 (-0.0513, -0.0158) | 0.0006 | 0.9735 (0.9330-1.0158) | 0.6109 |
| Free Cholesterol in Small VLDL, mmol/L | -0.0455 (-0.0634, -0.0277) | 2.52E-06 | 0.9682 (0.9280-1.0102) | 0.6109 |
| Triglycerides in Small VLDL, mmol/L | 0.0029 (-0.0145, 0.0204) | 0.7917 | 1.0044 (0.9629-1.0477) | 0.9399 |
| Concentration of Very Small VLDL Particles, mmol/L | -0.0120 (-0.0298, 0.0057) | 0.2496 | 1.0058 (0.9643-1.0490) | 0.9139 |
| Total Lipids in Very Small VLDL, mmol/L | -0.0073 (-0.0250, 0.0103) | 0.5132 | 1.0105 (0.9689-1.0539) | 0.8203 |
| Phospholipids in Very Small VLDL, mmol/L | -0.0012 (-0.0189, 0.0167) | 0.9065 | 1.0114 (0.9700-1.0545) | 0.7884 |
| Cholesterol in Very Small VLDL, mmol/L | -0.0225 (-0.0397, -0.0052) | 0.0212 | 1.0022 (0.9596-1.0466) | 0.9776 |
| Cholesteryl Esters in Very Small VLDL, mmol/L | -0.0257 (-0.0428, -0.0086) | 0.0079 | 1.0013 (0.9583-1.0463) | 0.9852 |
| Free Cholesterol in Very Small VLDL, mmol/L | -0.0140 (-0.0317, 0.0036) | 0.1777 | 1.0040 (0.9623-1.0475) | 0.9423 |
| Triglycerides in Very Small VLDL, mmol/L | 0.0234 (0.0057, 0.0412) | 0.0202 | 1.0238 (0.9828-1.0664) | 0.6109 |
| Concentration of IDL Particles, mmol/L | -0.0389 (-0.0565, -0.0214) | 4.57E-05 | 0.9812 (0.9399-1.0243) | 0.6621 |
| Total Lipids in IDL, mmol/L | -0.0379 (-0.0549, -0.0208) | 4.59E-05 | 0.9855 (0.9429-1.0300) | 0.7119 |
| Phospholipids in IDL, mmol/L | -0.0351 (-0.0522, -0.0180) | 0.0002 | 0.9872 (0.9445-1.0318) | 0.7615 |
| Cholesterol in IDL, mmol/L | -0.0435 (-0.0605, -0.0265) | 2.32E-06 | 0.9804 (0.9378-1.0250) | 0.6621 |
| Cholesteryl Esters in IDL, mmol/L | -0.0424 (-0.0594, -0.0255) | 3.81E-06 | 0.9815 (0.9388-1.0261) | 0.6676 |
| Free Cholesterol in IDL, mmol/L | -0.0455 (-0.0626, -0.0285) | 8.97E-07 | 0.9780 (0.9356-1.0224) | 0.6283 |
| Triglycerides in IDL, mmol/L | 0.0272 (0.0094, 0.0451) | 0.0069 | 1.0276 (0.9869-1.0701) | 0.6109 |
| Concentration of Large LDL Particles, mmol/L | -0.0474 (-0.0653, -0.0296) | 9.69E-07 | 0.9697 (0.9278-1.0135) | 0.6109 |
| Total Lipids in Large LDL, mmol/L | -0.0531 (-0.0708, -0.0354) | 3.51E-08 | 0.9698 (0.9291-1.0123) | 0.6109 |
| Phospholipids in Large LDL, mmol/L | -0.0543 (-0.0720, -0.0367) | 1.79E-08 | 0.9681 (0.9274-1.0106) | 0.6109 |
| Cholesterol in Large LDL, mmol/L | -0.0571 (-0.0747, -0.0395) | 3.67E-09 | 0.9667 (0.9260-1.0093) | 0.6109 |
| Cholesteryl Esters in Large LDL, mmol/L | -0.0558 (-0.0735, -0.0380) | 8.68E-09 | 0.9676 (0.9270-1.0100) | 0.6109 |
| Free Cholesterol in Large LDL, mmol/L | -0.0592 (-0.0766, -0.0418) | 7.05E-10 | 0.9652 (0.9239-1.0083) | 0.6109 |
| Triglycerides in Large LDL, mmol/L | 0.0241 (0.0063, 0.0419) | 0.0177 | 1.0217 (0.9810-1.0641) | 0.6109 |
| Concentration of Medium LDL Particles, mmol/L | -0.0472 (-0.0650, -0.0294) | 1.03E-06 | 0.9761 (0.9356-1.0183) | 0.6109 |
| Total Lipids in Medium LDL, mmol/L | -0.0505 (-0.0684, -0.0326) | 1.97E-07 | 0.9676 (0.9276-1.0094) | 0.6109 |
| Phospholipids in Medium LDL, mmol/L | -0.0520 (-0.0699, -0.0342) | 8.34E-08 | 0.9684 (0.9268-1.0118) | 0.6109 |
| Cholesterol in Medium LDL, mmol/L | -0.0541 (-0.0719, -0.0362) | 3.00E-08 | 0.9662 (0.9262-1.0080) | 0.6109 |
| Cholesteryl Esters in Medium LDL, mmol/L | -0.0492 (-0.0670, -0.0314) | 3.68E-07 | 0.9709 (0.9307-1.0128) | 0.6109 |
| Free Cholesterol in Medium LDL, mmol/L | -0.0663 (-0.0837, -0.0490) | 1.11E-11 | 0.9610 (0.9195-1.0044) | 0.6109 |
| Triglycerides in Medium LDL, mmol/L | 0.0189 (0.0013, 0.0366) | 0.0586 | 1.0148 (0.9741-1.0572) | 0.6926 |
| Concentration of Small LDL Particles, mmol/L | -0.0407 (-0.0585, -0.0229) | 2.67E-05 | 0.9774 (0.9370-1.0196) | 0.6109 |
| Total Lipids in Small LDL, mmol/L | -0.0451 (-0.0630, -0.0273) | 3.07E-06 | 0.9679 (0.9279-1.0096) | 0.6109 |
| Phospholipids in Small LDL, mmol/L | -0.0398 (-0.0577, -0.0218) | 4.59E-05 | 0.9693 (0.9294-1.0109) | 0.6109 |
| Cholesterol in Small LDL, mmol/L | -0.0521 (-0.0699, -0.0342) | 7.83E-08 | 0.9703 (0.9286-1.0138) | 0.6109 |
| Cholesteryl Esters in Small LDL, mmol/L | -0.0470 (-0.0648, -0.0292) | 1.08E-06 | 0.9680 (0.9279-1.0099) | 0.6109 |
| Free Cholesterol in Small LDL, mmol/L | -0.0596 (-0.0775, -0.0418) | 1.39E-09 | 0.9656 (0.9243-1.0088) | 0.6109 |
| Triglycerides in Small LDL, mmol/L | 0.0177 (0.0002, 0.0352) | 0.0755 | 1.0149 (0.9738-1.0576) | 0.6926 |
| Concentration of Very Large HDL Particles, mmol/L | 0.0108 (-0.0047, 0.0263) | 0.2374 | 1.0287 (0.9798-1.0802) | 0.6109 |
| Total Lipids in Very Large HDL, mmol/L | 0.0155 (4.35E-05, 0.0309) | 0.0786 | 1.0323 (0.9830-1.0841) | 0.6109 |
| Phospholipids in Very Large HDL, mmol/L | 0.0195 (0.0040, 0.0349) | 0.0259 | 1.0371 (0.9877-1.0889) | 0.6109 |
| Cholesterol in Very Large HDL, mmol/L | 0.0087 (-0.0067, 0.0240) | 0.3533 | 1.0230 (0.9738-1.0747) | 0.6565 |
| Cholesteryl Esters in Very Large HDL, mmol/L | 0.0061 (-0.0092, 0.0213) | 0.5313 | 1.0205 (0.9709-1.0726) | 0.6803 |
| Free Cholesterol in Very Large HDL, mmol/L | 0.0182 (0.0022, 0.0342) | 0.0436 | 1.0305 (0.9831-1.0801) | 0.6109 |
| Triglycerides in Very Large HDL, mmol/L | 0.0237 (0.0055, 0.0419) | 0.0212 | 1.0303 (0.9903-1.0719) | 0.6109 |
| Concentration of Large HDL Particles, mmol/L | 0.0057 (-0.0093, 0.0209) | 0.5466 | 1.0296 (0.9796-1.0822) | 0.6109 |
| Total Lipids in Large HDL, mmol/L | 0.0099 (-0.0053, 0.0251) | 0.2730 | 1.0342 (0.9843-1.0866) | 0.6109 |
| Phospholipids in Large HDL, mmol/L | 0.0143 (-0.0011, 0.0296) | 0.1063 | 1.0400 (0.9904-1.0921) | 0.6109 |
| Cholesterol in Large HDL, mmol/L | 0.0045 (-0.0106, 0.0196) | 0.6425 | 1.0248 (0.9748-1.0774) | 0.6385 |
| Cholesteryl Esters in Large HDL, mmol/L | 0.0024 (-0.0127, 0.0175) | 0.7938 | 1.0214 (0.9715-1.0739) | 0.6676 |
| Free Cholesterol in Large HDL, mmol/L | 0.0117 (-0.0035, 0.0269) | 0.1926 | 1.0361 (0.9860-1.0888) | 0.6109 |
| Triglycerides in Large HDL, mmol/L | 0.0237(0.0059-0.0415) | 0.0196 | 1.0293 (0.9861-1.0743) | 0.6109 |
| Concentration of Medium HDL Particles, mmol/L | -0.0015 (-0.0180, 0.0151) | 0.8847 | 1.0243 (0.9796-1.0710) | 0.6109 |
| Total Lipids in Medium HDL, mmol/L | 0.0035 (-0.0133, 0.0203) | 0.7458 | 1.0289 (0.9849-1.0749) | 0.6109 |
| Phospholipids in Medium HDL, mmol/L | 0.0094 (-0.0076, 0.0264) | 0.3584 | 1.0345 (0.9910-1.0799) | 0.6109 |
| Cholesterol in Medium HDL, mmol/L | -0.0047 (-0.0211, 0.0118) | 0.6536 | 1.0184 (0.9733-1.0655) | 0.6803 |
| Cholesteryl Esters in Medium HDL, mmol/L | -0.0058(-0.0223, 0.0106) | 0.5785 | 1.0158 (0.9710-1.0627) | 0.6993 |
| Free Cholesterol in Medium HDL, mmol/L | 0.0001 (-0.0161, 0.0164) | 0.9862 | 1.0281 (0.9824-1.0759) | 0.6109 |
| Triglycerides in Medium HDL, mmol/L | 0.0224 (0.0045, 0.0402) | 0.0267 | 1.0225 (0.9800-1.0668) | 0.6109 |
| Concentration of Small HDL Particles, mmol/L | -0.0436 (-0.0617, -0.0254) | 9.65E-06 | 0.9782 (0.9386-1.0195) | 0.6109 |
| Total Lipids in Small HDL, mmol/L | -0.0217 (-0.0397, -0.0037) | 0.0334 | 0.9985 (0.9584-1.0403) | 0.9852 |
| Phospholipids in Small HDL, mmol/L | -0.0109 (-0.0289, 0.0071) | 0.3105 | 1.0086 (0.9682-1.0507) | 0.8577 |
| Cholesterol in Small HDL, mmol/L | -0.0437 (-0.0619, -0.0255) | 9.45E-06 | 0.9783 (0.9387-1.0195) | 0.6109 |
| Cholesteryl Esters in Small HDL, mmol/L | -0.0485 (-0.0668, -0.0303) | 9.69E-07 | 0.9719 (0.9326-1.0128) | 0.6109 |
| Free Cholesterol in Small HDL, mmol/L | -0.0233 (-0.0411, -0.0054) | 0.0212 | 1.0007 (0.9601-1.0429) | 0.9935 |
| Triglycerides in Small HDL, mmol/L | 0.0146 (-0.0023, 0.0316) | 0.1397 | 1.0189 (0.9760-1.0637) | 0.6621 |

Lung function was dichotomous and the normal level was considered as the reference group. Metabolites concentrations were standardized by z-score.

^a^Models were adjusted for age, sex, education, employment, assessment center, Townsend deprivation index, height, smoking status, drinking status,

physical activity, BMI, sleep duration, hypertension, diabetes, heart failure, stroke, coronary heart disease, ischemic heart disease, and lung-related diseases. ^b^Models were adjusted for age, sex, education, employment, assessment center, Townsend deprivation index, height, smoking status, drinking status, physical activity, BMI, sleep duration, hypertension, diabetes, heart failure, stroke, coronary heart disease, ischemic heart disease, lung-related diseases, and lung function. Bolded indicated that biomarkers may be potential mediators in the association between lung function and the risk of depression. FDR, false discovery rate; PM, proportion mediated; CI, confidence interval; HR, hazard ratio.


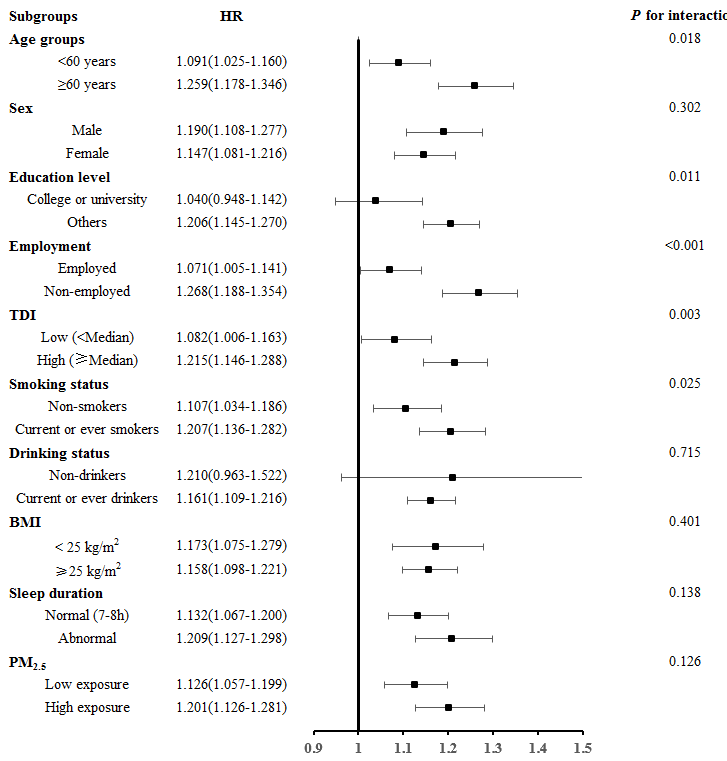


Fig. S1. Subgroup analysis of the association of lung function on depression by potential risk factors.

Abbreviations: confidence interval; HR, hazard ratio; TDI, Townsend deprivation index; BMI, body mass index. Models were adjusted for age at baseline, sex, education, employment, assessment center, Townsend deprivation index, height, smoking status, drinking status, physical activity, BMI, sleep duration, hypertension, diabetes, heart failure, stroke, coronary heart disease, ischemic heart disease, and pre-existing lung diseases.
